# IMp: The customizable LEGO^®^ Pinned Insect Manipulator

**DOI:** 10.3897/zookeys.481.8788

**Published:** 2015-02-04

**Authors:** Steen Dupont, Benjamin Price, Vladimir Blagoderov

**Affiliations:** 1 Department of Life Sciences, Natural History Museum, London, SW7 5BD, U.K.; 2 Department of Science Facilities, Natural History Museum, London, SW7 5BD, U.K.

**Keywords:** Specimen Manipulator, Entomology, Stage, Digitization, Imaging

## Abstract

We present a pinned insect manipulator (IMp) constructed of LEGO® building bricks with two axes of movement and two axes of rotation. In addition we present three variants of the IMp to emphasise the modular design, which facilitates resizing to meet the full range of pinned insect specimens, is fully customizable, collapsible, affordable and does not require specialist tools or knowledge to assemble.

## Introduction

Natural history collections are one of the most fundamentally important institutions in science, where voucher specimens are housed in perpetuity, embodying the research of generations of scientists. Within entomology collections insects were historically preserved by drying on an appropriately sized pin. Today insect groups are preserved in ethanol, mounted on microscope slides or in paper or plastic envelopes, but the dry pinned method has not changed for the majority of insect orders since its development in the early 18th century. This has resulted in millions of pinned insect specimens housed in natural history collections globally, for example the pinned insect collection of the Natural History Museum (NHM) in London comprises 136500 drawers and is estimated to contain 27 million pinned specimens.

Although pinned specimens preserve well they become fragile with age and are prone to damage when handled. This has become especially apparent in the digital age where there is an increased focus on mobilizing the vast amounts of biodiversity data stored in the collections through digitization activities. Institutions are now able to provide images of specimens on request (termed a “digital loan” at the NHM), when researchers cannot view the specimens directly and they are too fragile to ship. In addition some institutions do not loan primary type material, compounding the need to image specimens in order to examine them remotely. The net result of improving access to collections through digitization efforts is that the specimens are made available without the need of excessive handling. This goal however can only be achieved when there are images of multiple taxonomically meaningful views available, as required for accurate remote examination.

Most commonly, pinned insect specimens are observed through stereo microscopes at the 10-80× range of magnification. Under magnification specimens can become difficult to handle as minute movements are amplified. In order to provide stabilization specimens are often pinned to a flexible material such as cork, plastazote foam or putty when the desired angle is found or alternatively the specimen is mounted into a specimen manipulator that enables repositioning during observation.

With the rapid increase in collections digitization, museum specimens are handled to a much larger extent than ever before. Positioning and repositioning of specimens during digitization is often required for the majority of specimen handling in a collection. As handling of pinned specimens carries the most immediate risk of damage, especially to the fragile extremities (e.g. legs, antenna and wings) specimen manipulators are of great value to the overall preservation of a functioning pinned natural history collection.

A good insect specimen manipulator requires the following properties: (i) Foremost the manipulator should allow for easy positioning and repositioning of specimens especially if used for imaging at multiple views or comparing structures at different angles; (ii) Stability to prevent the specimen moving once in the correct position; (iii) Capability of fine scale adjustment to enable positioning the specimen under magnification and (iv) Open design to allow for both specimen placement/removal and adequate illumination of the specimen to be examined.

There are several good designs available from commercial and amateur DIY websites such as the Universal Stage (http://extreme-macro.co.uk/universal-stage/), Rose Entomology (http://www.roseentomology.com/Pinned_Specimen_Manipulator.htm), BioQuip Microscope Stages 6186 and 6188 (http://www.bioquip.com/search/DispProduct.asp?pid=6186; http://www.bioquip.com/search/DispProduct.asp?pid=6188) and the Watkins & Doncaster Insect Examination Stage (http://www.watdon.co.uk/acatalog/Microscope_Accessories.html). In addition there are variants of the steel / brass ball & ring stage combination (Ento / Ergo Ball: details available on request).

Previous authors have provided custom designs for insect specimen manipulators (Köppen 1966, Oliver 1969, Lobanov and Kotjurgin 1975, Boyadzhiev and Bozhinova 2006, Boyadzhiev et al. 2012), however most commercial examples are of a fixed standard size while DIY manipulators are custom-made from materials and tools that are not readily available to everyone. Furthermore most DIY setups are specifically designed for a particular group of insects and may not be of an appropriate size for other insect groups. We believe that the design presented here is a solution to an insect specimen manipulator that is (a) universally applicable, (b) readily available, (c) cost effective, (d) portable and (e) fully customizable.

## Material and methods

The idea of a holding mechanism for pinned specimens is as old as the pinned specimen itself. The design of these particular models were inspired by the daily grind of comparative morphology and the association to mass digitization and digital loans that the first author has had at the natural history museums of Denmark and London. Although the LEGO® brick has always been a working tool it has served more as a means of prototyping ideas, but in this case the authors found the plastic bricks to have the right properties for the product presented here. It is in fact the simple nature of the LEGO® bricks, their availability and ease of use that we feel make these models so customizable, user friendly, affordable and hassle free.

The Insect specimen manipulator (IMp) and subsequent size/design variants were built and designed using both the LEGO® building blocks and the LEGO® Digital designer software version 4.3.8 (http://ldd.lego.com/en-gb/) using beams, beam connectors, connecting pins, an 8 tooth spur gear and a worm gear. For a complete parts list and assembly manual for all IMp models see the supplementary information (http://dx.doi.org/10.5519/0036449).

## Data resources

The data underpinning the analysis reported in this paper are deposited in the NHM Data Portal at http://dx.doi.org/10.5519/0036449.

## Results and discussion

*Etymology*: IMp is an abbreviation of Insect Manipulator and references the attendant imp of folklore that is usually cast as the small, mischievous helper, associated with witches and warlocks, the academics of mythology.

**Figure 1.**
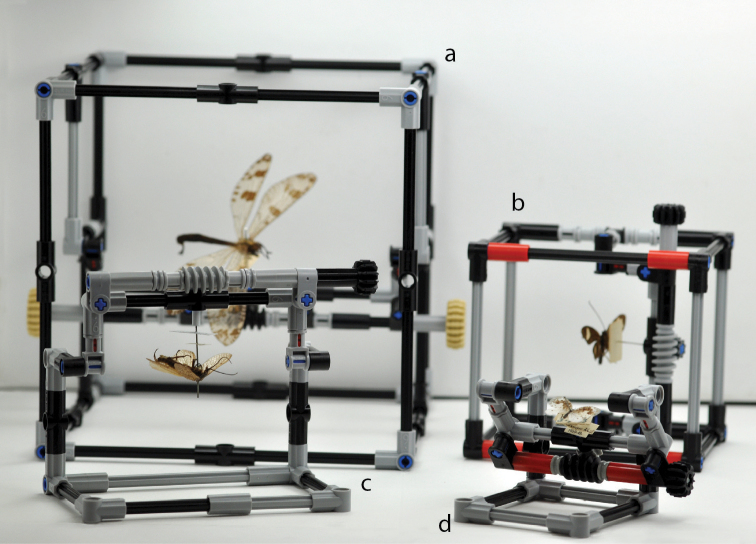
The four different sized manipulators shown for comparison: **a** Giant-IMp **b** IMp models with encasing support beams **c** Open-IMp **d** Micro-IMp models that are not encased. The specimens in the manipulators are: **a** *Nosa tristis* (Hagen, 1853) – Neuroptera: Myrmeleontidae **b** *Perissoneura paradoxa* McLachlan, 1871 – Trichoptera: Odontoceridae **c** *Pteronarcys californica* Newport, 1848 – Plecoptera: Pteronarcyidae and **d** *Psychopsis coelivaga* (Walker, 1853) – Neuroptera: Psychopsidae.

**Figure 2.**
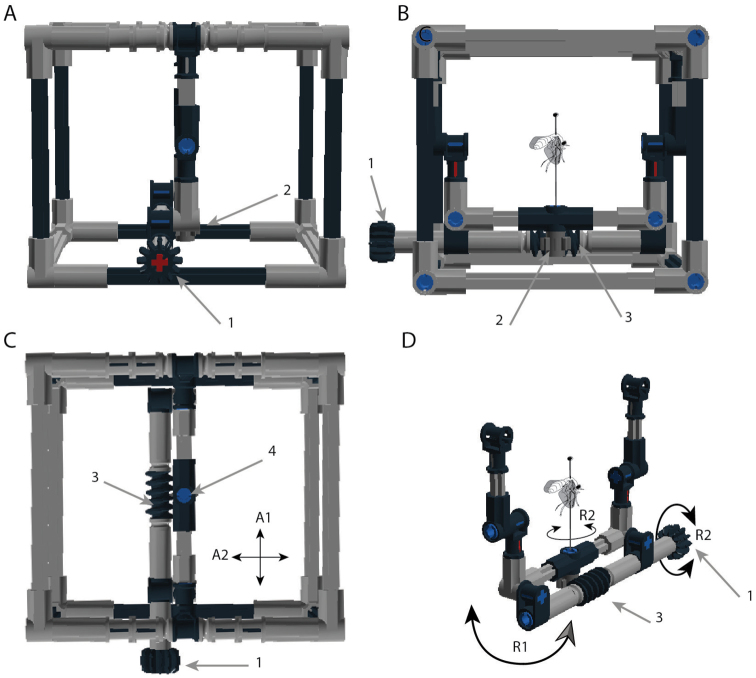
The IMp model shown from a side view (**A**), front view (**B**), top view (**C**) and without the stabilizing case exposing the pivot arm wherein the specimen is placed (**D**). Figure labels and abbreviations: pivot handle (**1**), 8 tooth gear (**2**), worm gear (**3**), connector peg that holds and rotates the specimen (**4**), axis of movement 1 (**A1**), axis of movement 2 (**A2**), axis of rotation 1 (**R1**) and axis of rotation 2 (**R2**).

Initially a single enclosed IMp design was conceived (Figures 1b, 2), capable of accommodating insect specimens up to 50 mm in length, with 5 mm clearance on either side of the specimen. Three subsequent models were then designed to facilitate the examination of insects of varying sizes, and to display the customizable nature of the IMp base design, the relative size of each can be seen in Figure 1. The design of the IMp model on which all subsequent variants are based, and the axes of movement and rotation, are shown in more detail in Figure 2 and the Suppl. material 2. The original IMp and the Giant-IMp models are encased with support beams, adding stability to the design and physical protection for the specimens, whereas the Micro-IMp and Open-IMp models are of an open design that allows for a smaller working distance between the specimen and the stereo microscope. The size and cost (excluding shipping) of each model is summarized in Table 1.

**Table 1.** Summary of the features of each of the IMp models.

Model name	Maximum specimen size^1^ (mm)	Cost^2^ (£)	Design^3^
IMp	50	9	closed^4^
Micro-	30	7	open
Open-	60	8	open
Giant-	110	15	closed^4^

1 allowing 5 mm clearance on either side;

2 cost (rounded to nearest pound) at time of publication and excluding shipping;

3 open design facilitates a closer working distance, while the closed design includes the supporting cube structure for stability and additional specimen protection;

4 supports can be removed if a closer working distance is required.

**Figure 3.**
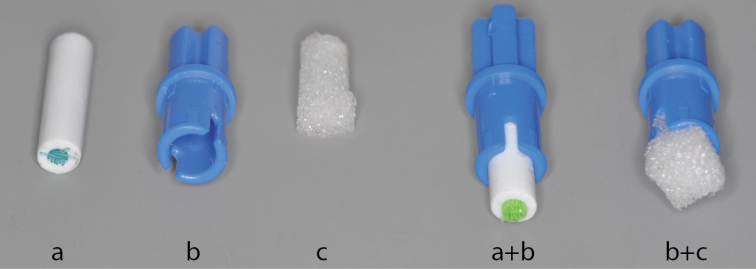
Two options for the modification to the connector pegs (**b**) to allow for insertion of the specimen pin: a 3 mm diameter tube with 0.1 mm nylon fibers (**a+b**); or a small plastazote plug (**b+c**).

**Figure 4.**
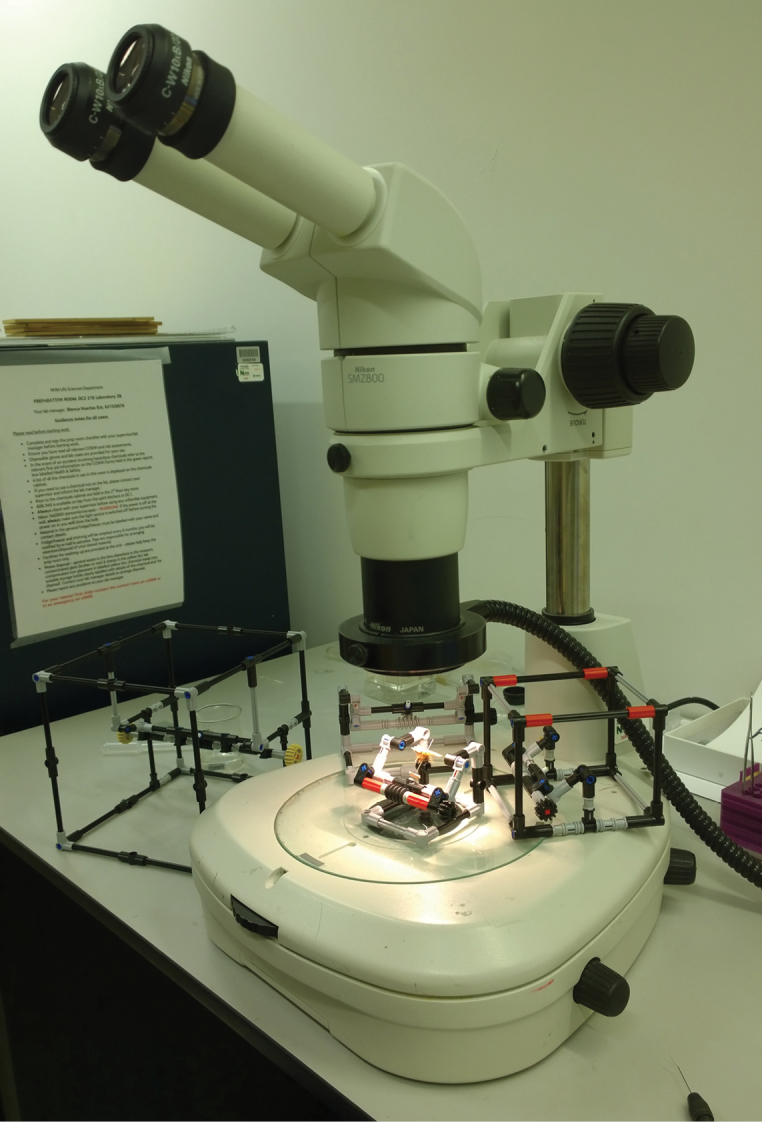
The IMp models being used with the micro-IMp holding a specimen positioned for viewing.

The bricks for all models can be bought directly from the LEGO® websites’ Pick a Brick (http://shop.lego.com/en-GB/Pick-A-Brick-ByTheme) and the Bricks and Pieces selection (https://service.lego.com/en-gb/replacementparts#BasicInfo). Besides the LEGO® bricks the models each require a small plastazote foam / cork plug or other material that will allow for the pin to be held in place (Figure 3). The models presented here all use a 3 mm × 10 mm plastic tube with nylon toothbrush bristles in the center. This allows for any size pin to be held firmly in place while allowing repeated use without the degradation that is common when repeatedly pinning into foam or cork inserts over time. Except for the Giant-IMp the smaller versions are small enough to fit under a standard stereo microscope (Figure 4). The Giant-IMp however was designed for use with large specimens that are usually imaged using a standard DSLR setup with a much larger working distance.

We believe the insect specimen manipulators presented here are a valuable addition to any entomologist’s toolbox and that the use of any insect manipulator is in the interest of anyone dealing with valuable specimens as the actual handling of the specimen is reduced to a minimum during examination. In case of the original IMp and Giant-IMp models the specimens are further protected from accidental contact during examination by the supporting cube structure. These LEGO® based manipulators benefit from their modular design as they are inexpensive and made from readily available components. Furthermore, even the largest of the models can be disassembled for travel. The open design further allows for the addition of portable lighting solutions (such as LEDs) and an endless amount of customization which makes them ideal for specimen imaging. Future modifications of the IMp models may include the addition of motorized control, using Arduino controllers or native LEGO® motors and software from the LEGO® mindstorms range.

The authors welcome correspondence on ideas for the next generation of IMps, and although the current models are easy to assemble the authors are happy to assist if no children can be sourced locally.

## Supplementary materials

Supplementary material 1 Annotated building instructions Data type: Adobe PDF file. Explanation note: Assembly manual for all IMp models. This dataset is made available under the Open Database License (http://opendatacommons.org/licenses/odbl/1.0/). The Open Database License (ODbL) is a license agreement intended to allow users to freely share, modify, and use this Dataset while maintaining this same freedom for others, provided that the original source and author(s) are credited.Steen Dupont, Benjamin Price, Vladimir Blagoderov

Supplementary material 2 Video demontration Data type: MOV file. This dataset is made available under the Open Database License (http://opendatacommons.org/licenses/odbl/1.0/). The Open Database License (ODbL) is a license agreement intended to allow users to freely share, modify, and use this Dataset while maintaining this same freedom for others, provided that the original source and author(s) are credited.Steen Dupont, Benjamin Price, Vladimir Blagoderov
