# Supplementary material for: IMp: The customizable LEGO® Pinned Insect Manipulator
Source: Zookeys. 2015 Feb 4;(481):131–8. doi: 10.3897/zookeys.481.8788 (PMC4319104; doi:10.3897/zookeys.481.8788)
Supplement: Supplementary material 1 — Annotated building instructions [file zookeys-481-131-s001.pdf]

IMp: The customizable LEGO® Pinned Insect Manipulator

Steen Dupont, Benjamin Price*, Vladimir Blagoderov***

** Department of Life Sciences, Natural History Museum, London, SW7 5BD, U.K.*

*** Department of Science Facilities, Natural History Museum, London, SW7 5BD, U.K.*

Supplement 1: Annotated building instructions

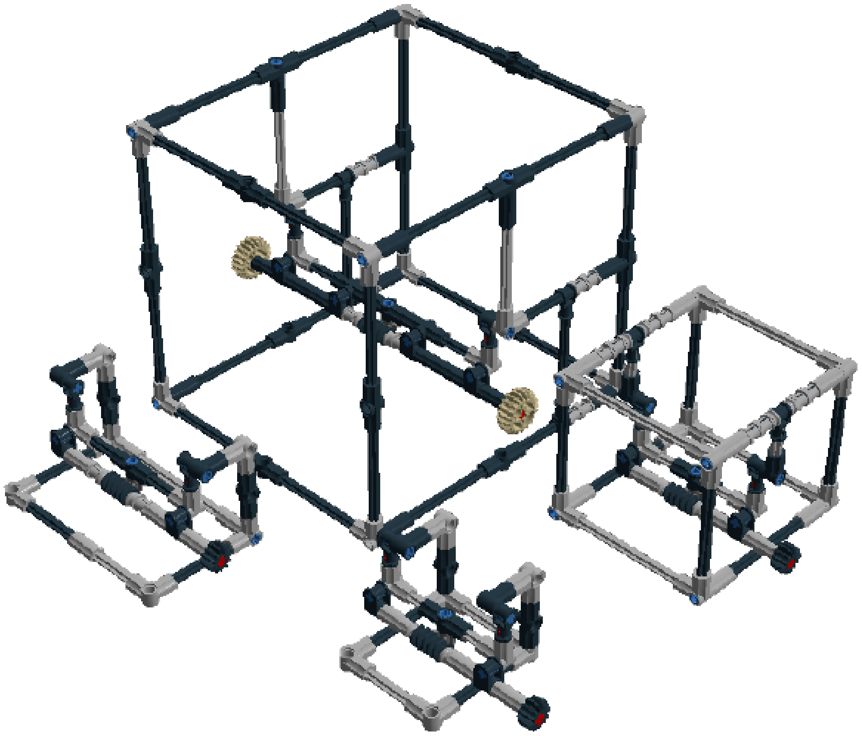

Table of Contents

Micro-IMp and parts list	2
Micro-IMp building instructions	3-14
IMp and parts list	15
IMp building instructions	16-30
Open-IMp and parts list	31
Open-IMp building instructions	33-46
Giant-IMp and parts list	47
Giant-IMp building instructions	59-70
Parts list for all IMp models	70-73

Building instructions[1] for the Micro-IMp
Number of bricks = 48

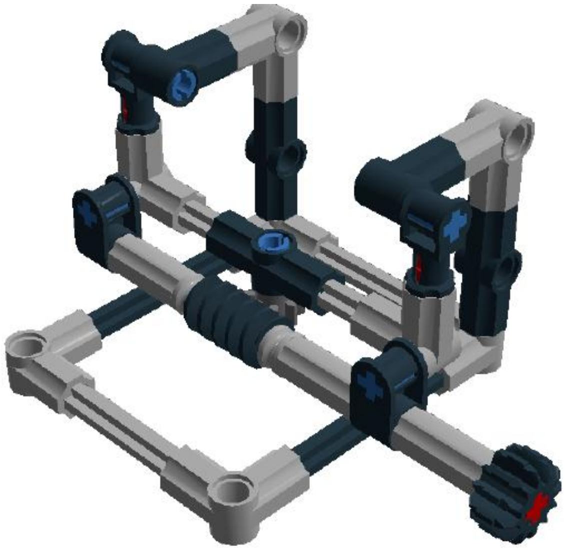

Brick assortment for the Micro-Imp
(for correct purchase codes see page 70-73)

2 x	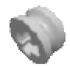	4211573	1/2 BUSH - Medium Stone Grey
7 x	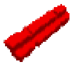	4142865	2M CROSS AXLE W. GROOVE - Bright Red
3 x	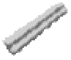	4211815	CROSS AXLE 3M - Medium Stone Grey
8 x	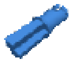	4225927	CONNECTOR PEG/CROSS AXLE - Bright Blue
3 x	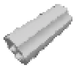	4512360	CROSS AXLE, EXTENSION, 2M - Medium Stone Grey
2 x	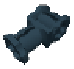	4107081	CATCH W. CROSS HOLE - Black
3 x	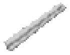	4211639	CROSS AXLE 5M - Medium Stone Grey
2 x	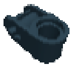	653626	CROSS BLOCK 90° - Black
2 x	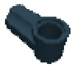	4107085	ANGLE ELEMENT, 0 DEGREES [1] - Black
2 x	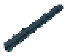	370626	CROSS AXLE 6M - Black
3 x	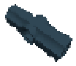	4107783	ANGLE ELEMENT, 180 DEGREES [2] - Black
8 x	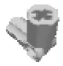	4211670	ANGLE ELEMENT, 90 DEGREES [6] - Medium Stone Grey
1 x	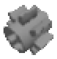	4514559	GEAR WHEEL T=8, M=1 - Dark Stone Grey
1 x	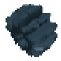	4177431	DOUBLE CONICAL WHEEL Z12 1M - Black
1 x	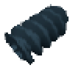	471626	WORM - Black

[1] As generated by LEGO® digital designer 4.3.8

Step 1 of 25

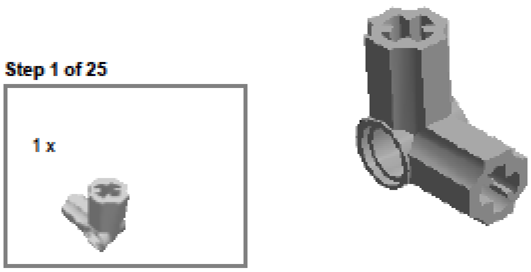

Step 2 of 25

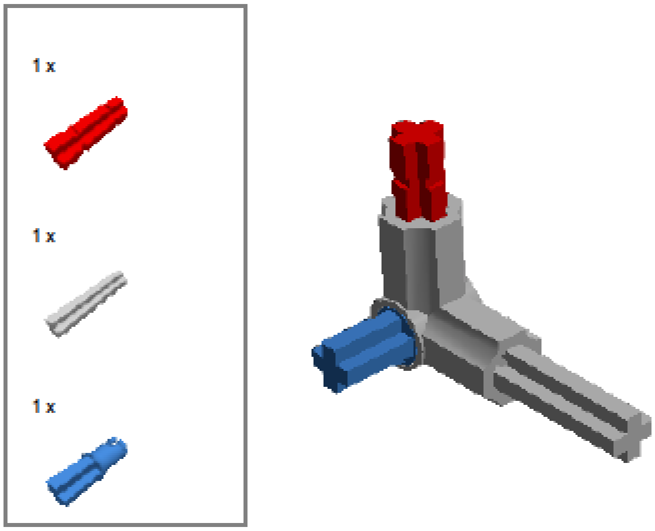

Step 3 of 25

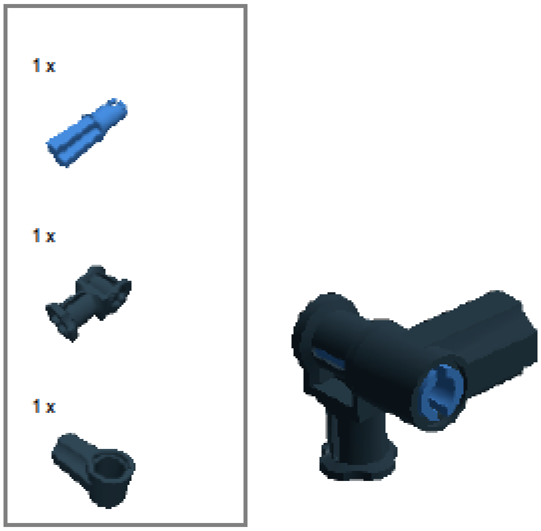

Step 4 of 25

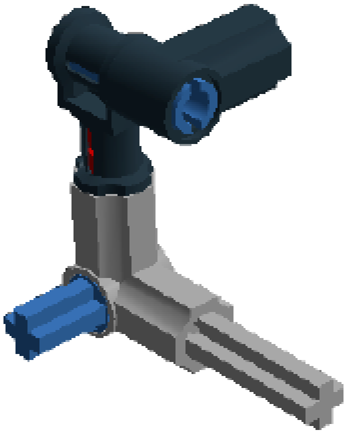

Step 5 of 25

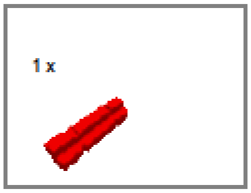

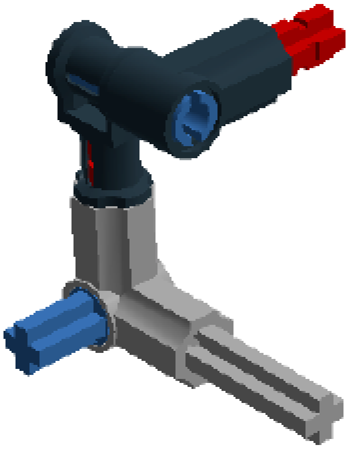

Step 6 of 25

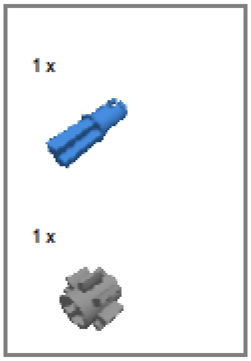

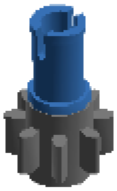

Step 7 of 25

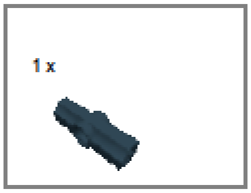

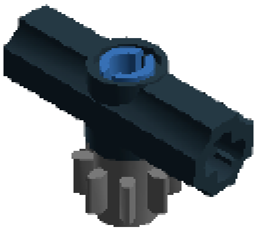

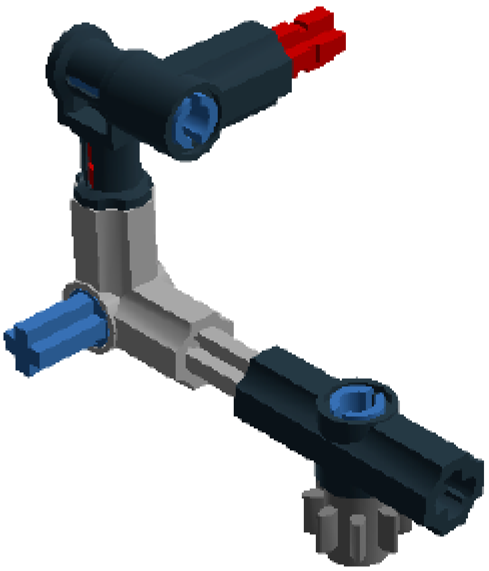

Step 8 of 25

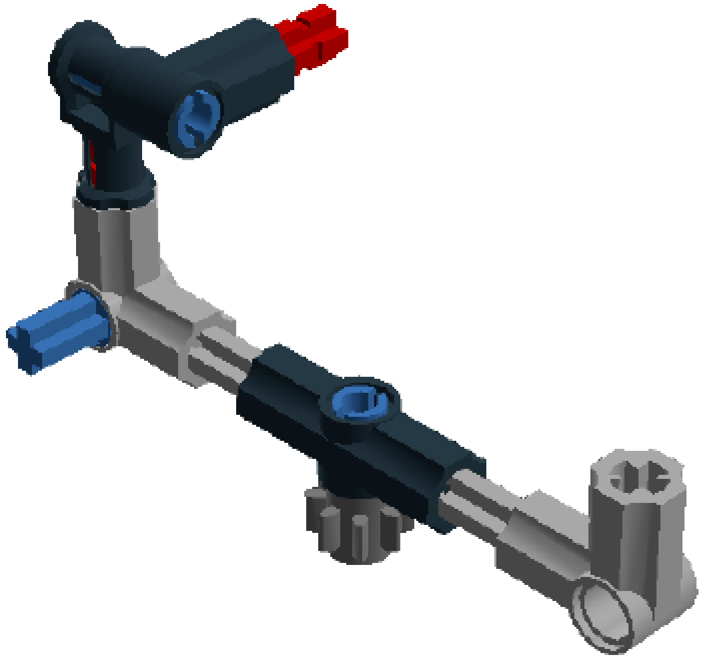

Step 9 of 25

1 x

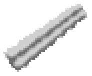

1 x

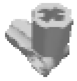

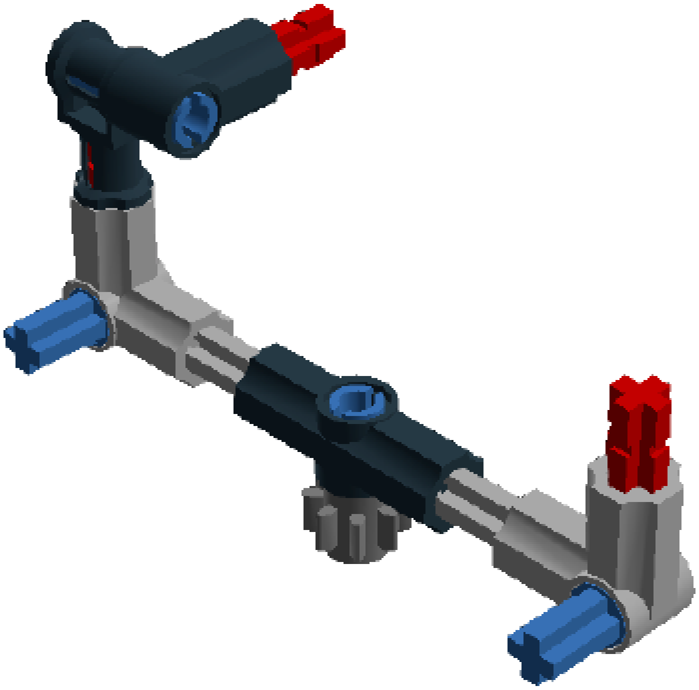

Step 10 of 25

1 x

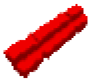

1 x

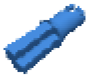

Step 11 of 25

1 x

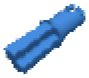

1 x

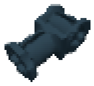

1 x

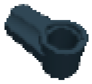

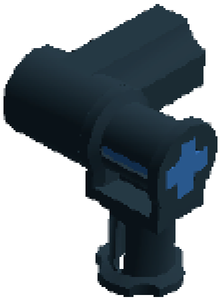

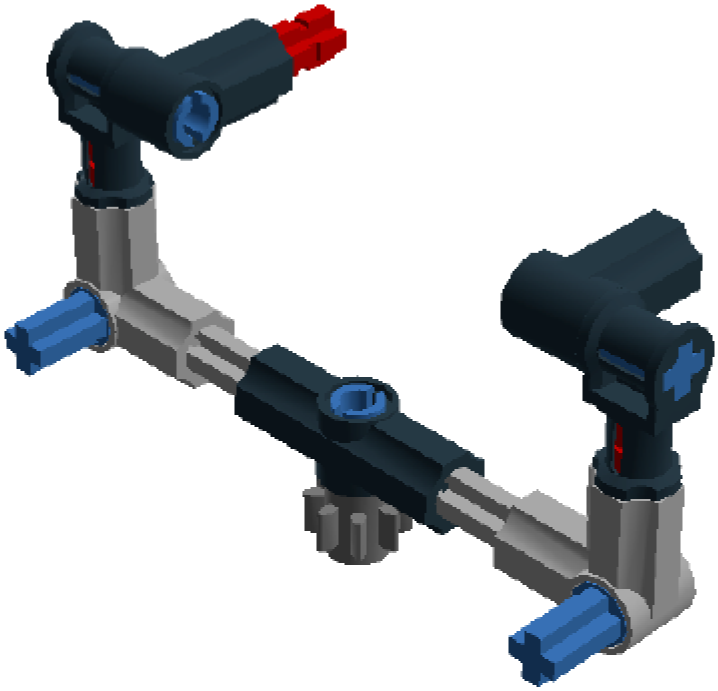

Step 12 of 25

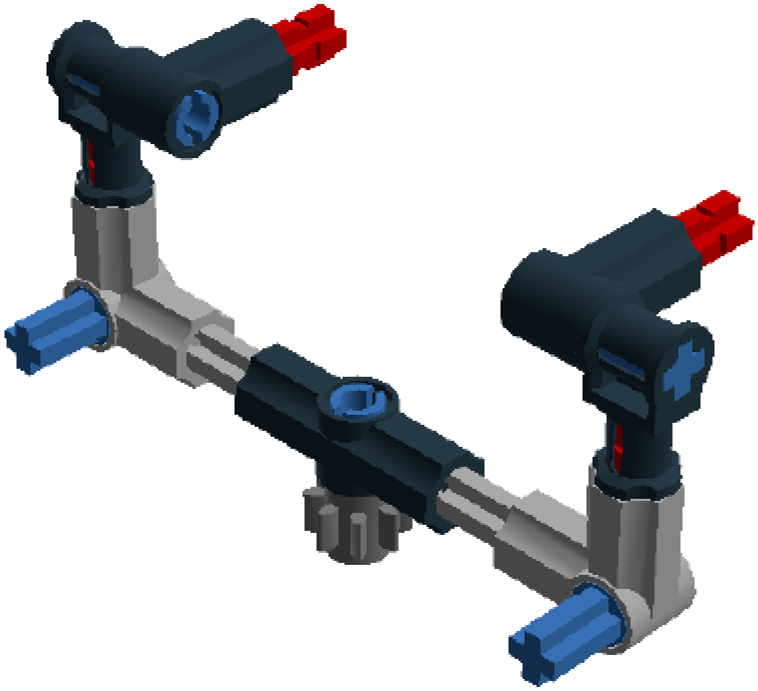

Step 13 of 25

1 x

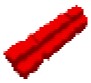

Step 14 of 25

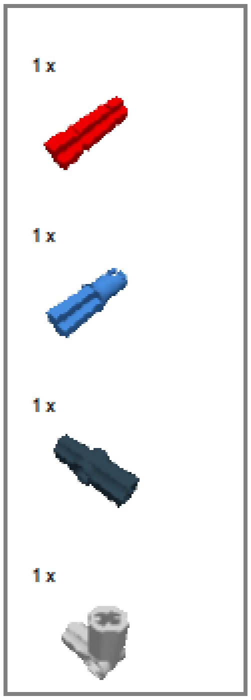

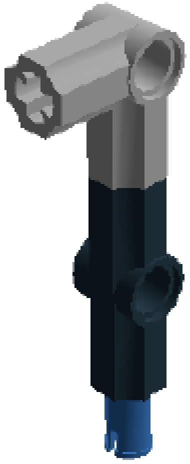

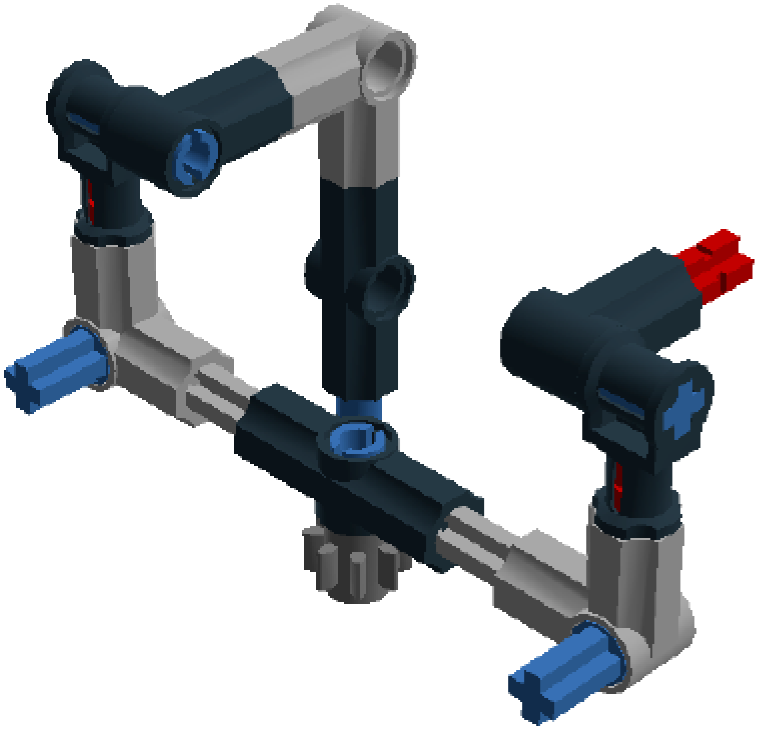

Step 15 of 25

Step 16 of 25

1 x

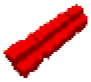

1 x

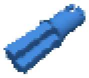

1 x

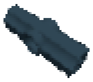

1 x

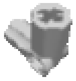

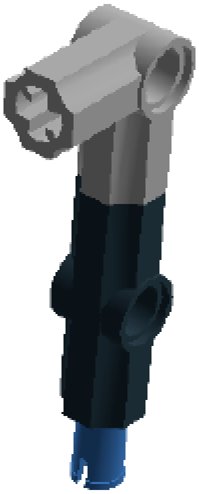

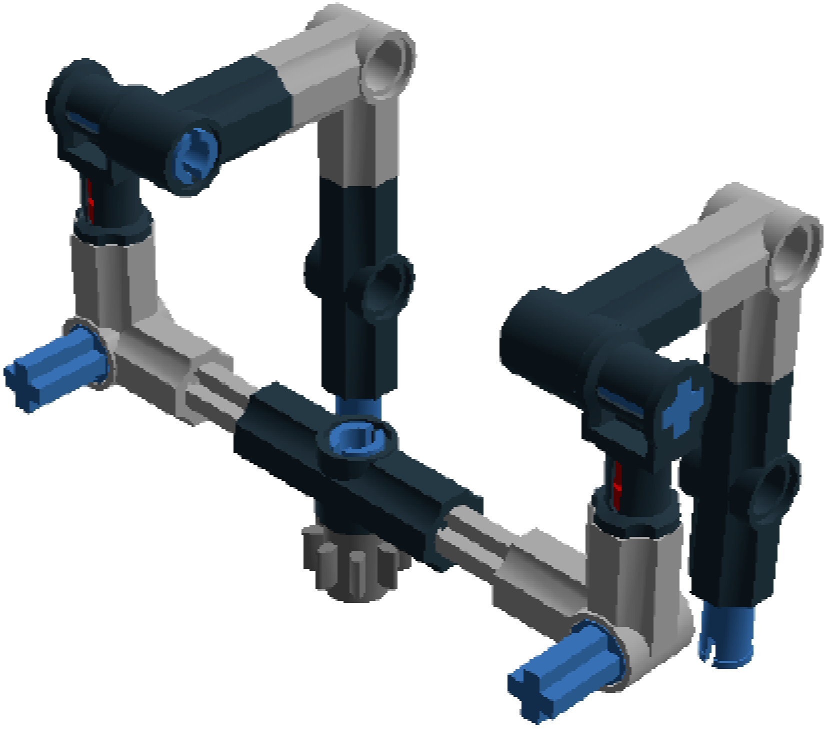

Step 17 of 25

Step 18 of 25

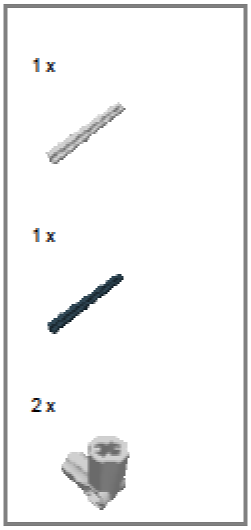

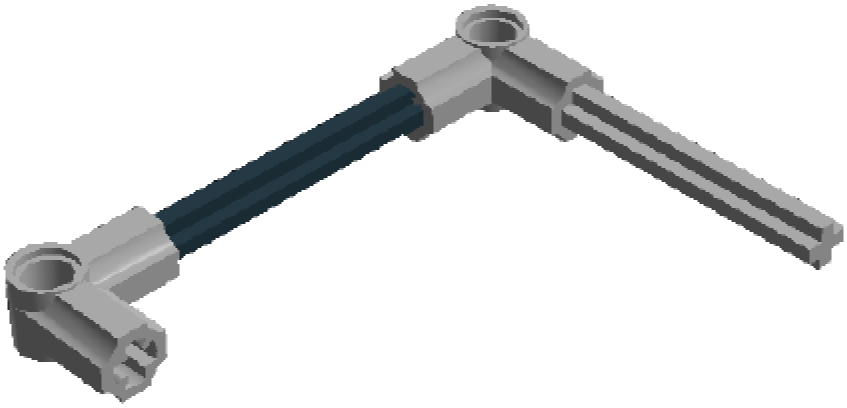

Step 19 of 25

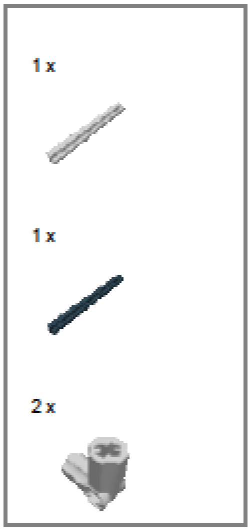

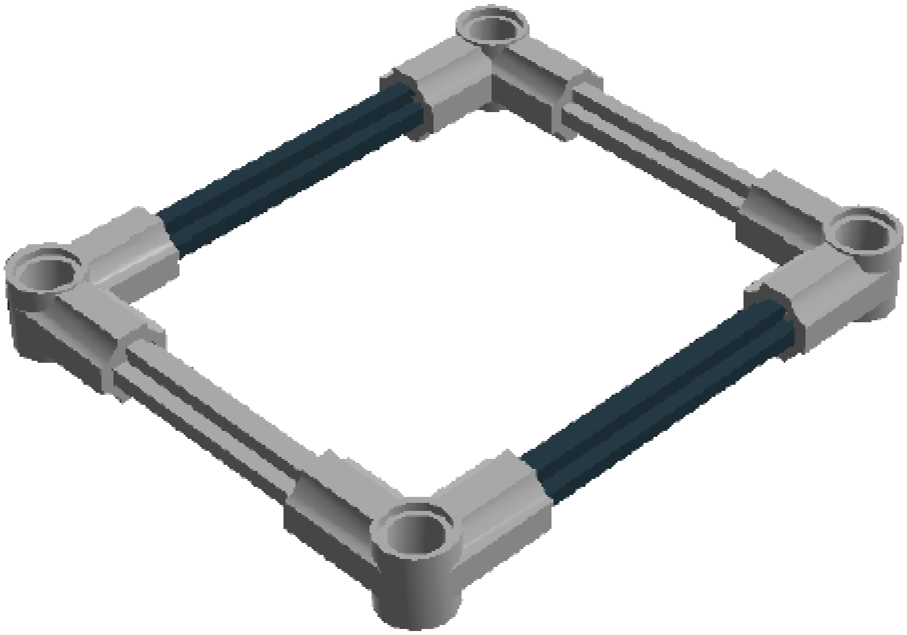

Step 20 of 25

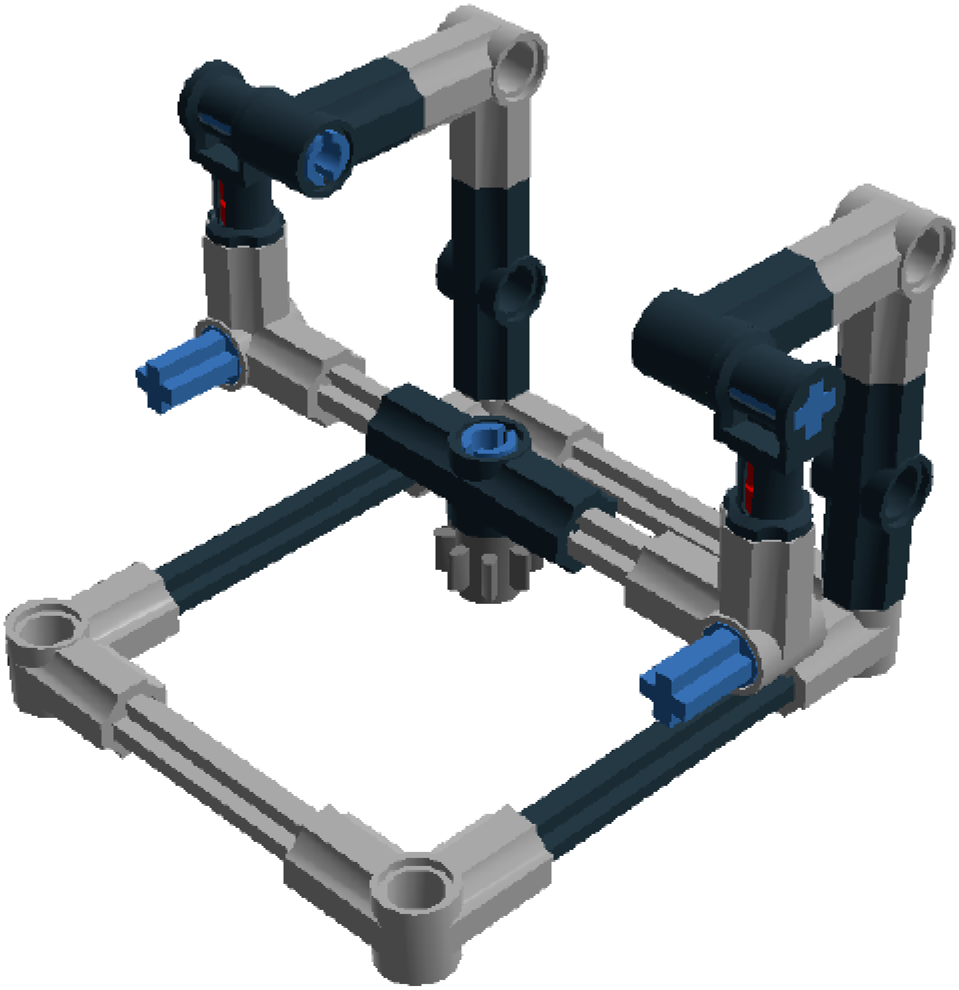

Step 21 of 25

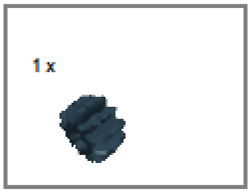

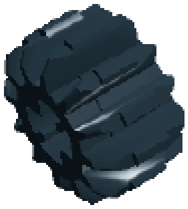

Step 22 of 25

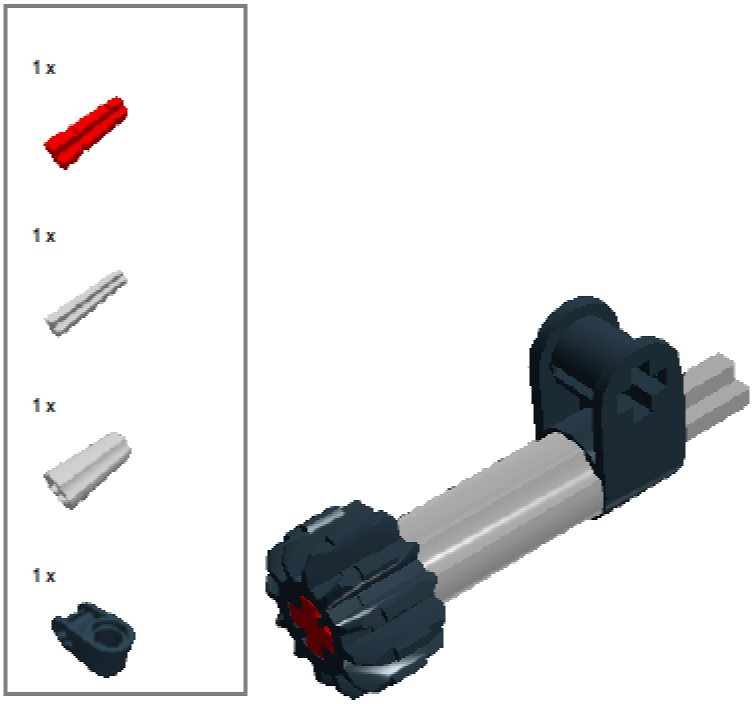

Step 23 of 25

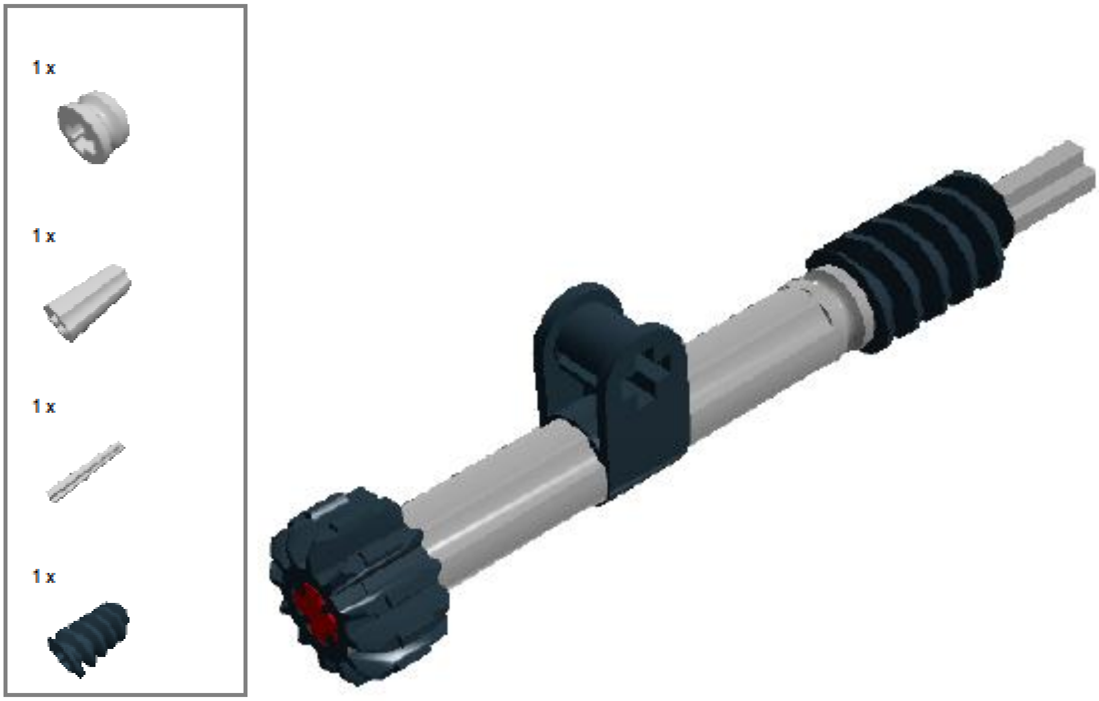

Step 24 of 25

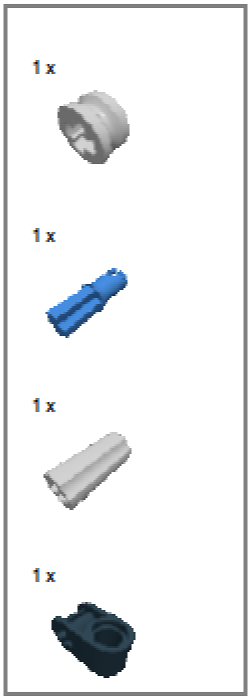

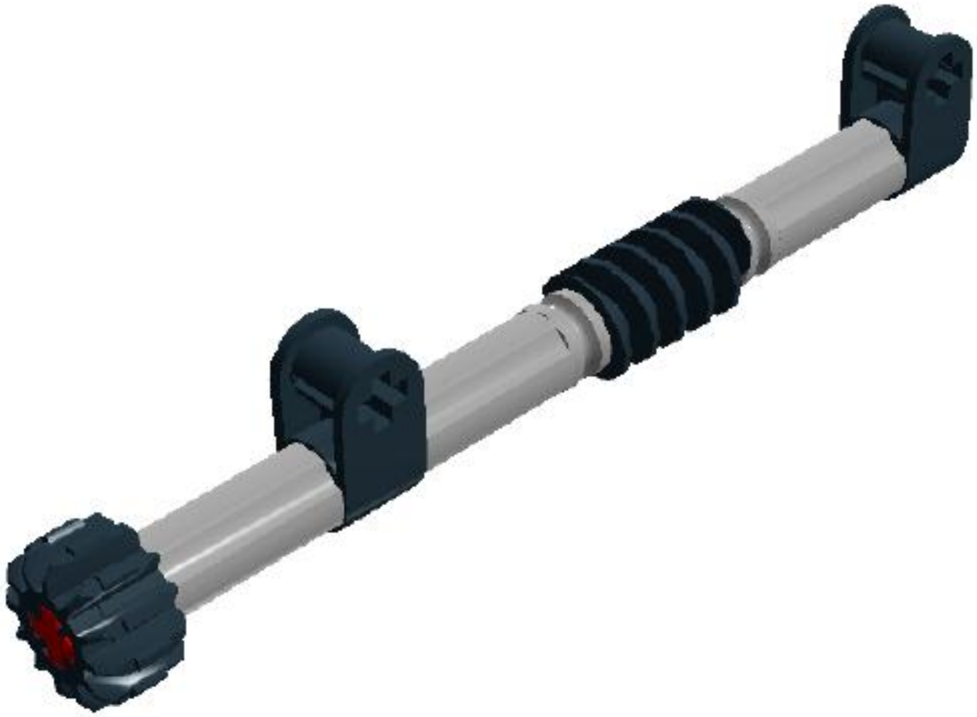

Step 25 of 25

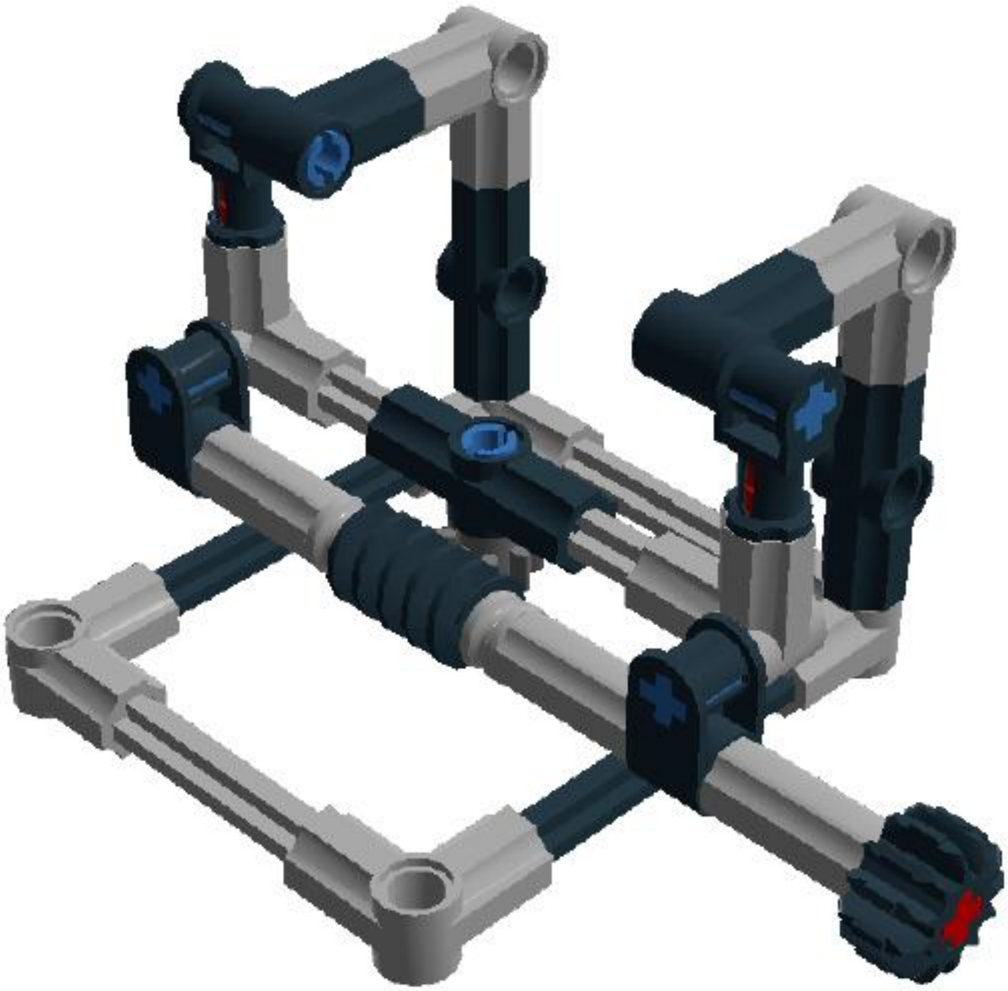

Building instructions[2] for the IMp
Number of bricks = 82

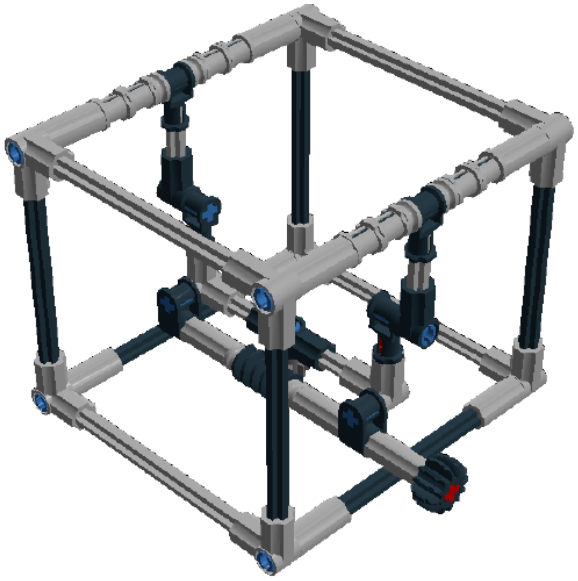

Brick assortment for the Imp
(for correct purchase codes see page 70-73)

6 x	4211573	1/2 BUSH - Medium Stone Grey	3 x	4142865	2M CROSS AXLE W. GROOVE - Bright Red	5 x	4211815	CROSS AXLE 3M - Medium Stone Grey
14 x	4225927	CONNECTOR PEG/CROSS AXLE - Bright Blue	8 x	4211622	BUSH FOR CROSS AXLE - Medium Stone Grey	11 x	4512360	CROSS AXLE, EXTENSION, 2M - Medium Stone Grey
4 x	4107081	CATCH W. CROSS HOLE - Black	1 x	4211639	CROSS AXLE 5M - Medium Stone Grey	2 x	653626	CROSS BLOCK 90° - Black
2 x	4107085	ANGLE ELEMENT, 0 DEGREES [1] - Black	8 x	370726	CROSS AXLE 8M - Black	1 x	4107783	ANGLE ELEMENT, 180 DEGREES [2] - Black
4 x	4535768	CROSS AXLE 9M - Medium Stone Grey	10 x	4211670	ANGLE ELEMENT, 90 DEGREES [6] - Medium Stone Grey	1 x	4514559	GEAR WHEEL T=8, M=1 - Dark Stone Grey
1 x	4177431	DOUBLE CONICAL WHEEL Z12 1M - Black	1 x	471626	WORM - Black			

[2] As generated by LEGO® digital designer 4.3.8

Step 1 of 37

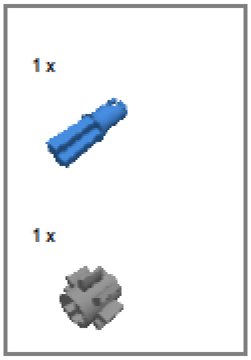

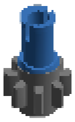

Step 2 of 37

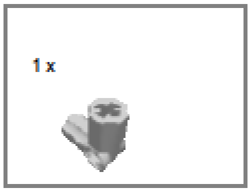

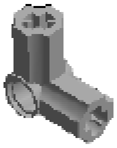

Step 3 of 37

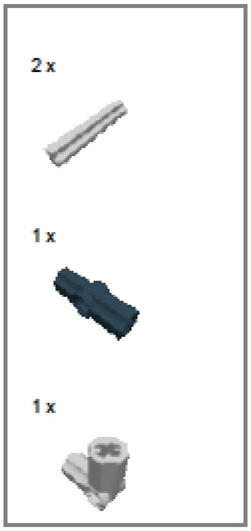

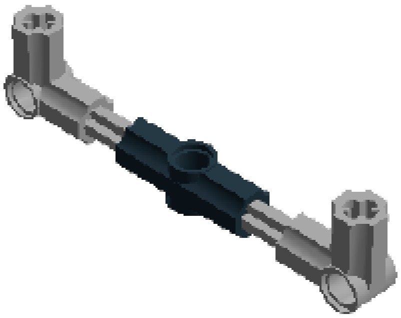

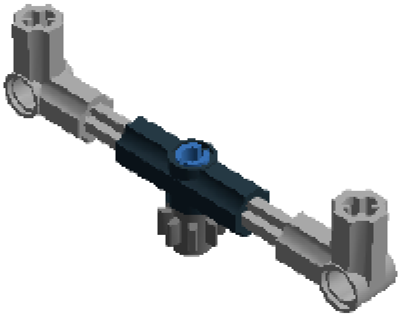

Step 4 of 37

Step 5 of 37

1 x

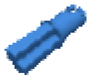

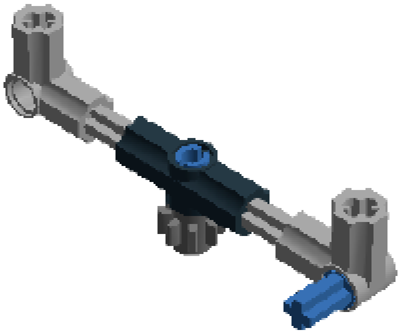

Step 6 of 37

2 x

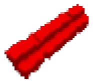

1 x

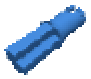

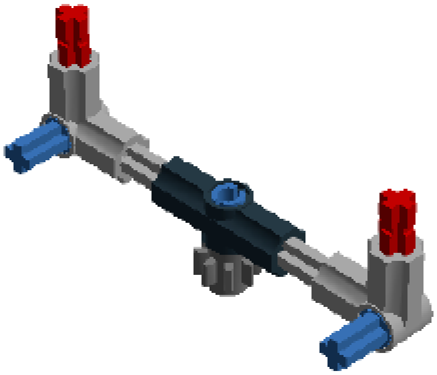

Step 7 of 37

1 x

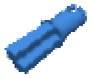

1 x

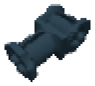

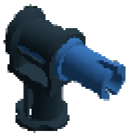

Step 8 of 37

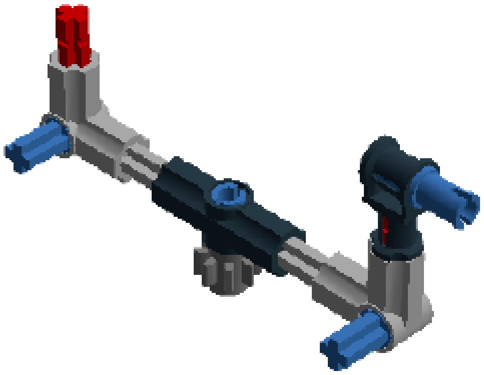

Step 9 of 37

1 x

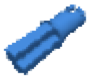

1 x

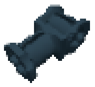

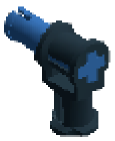

Step 10 of 37

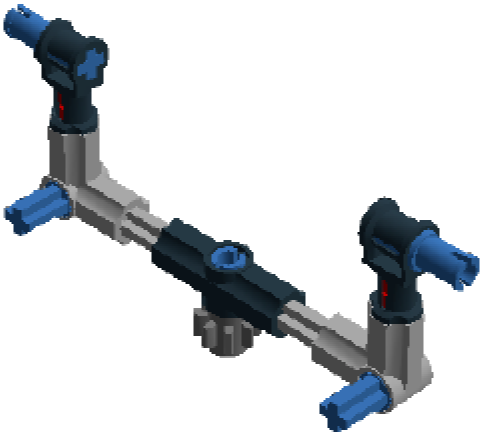

Step 11 of 37

1 x

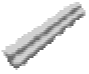

1 x

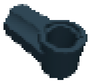

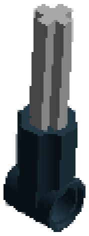

Step 12 of 37

1 x

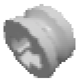

1 x

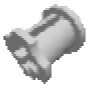

1 x

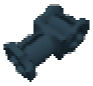

1 x

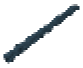

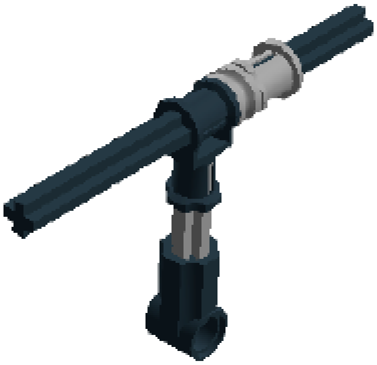

Step 13 of 37

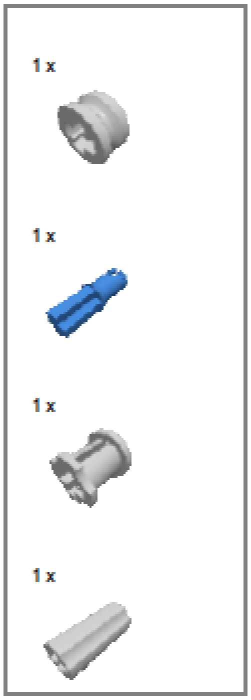

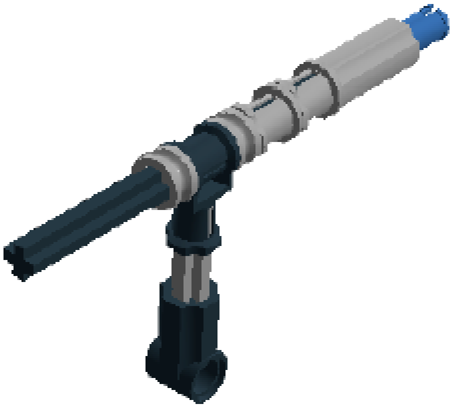

Step 14 of 37

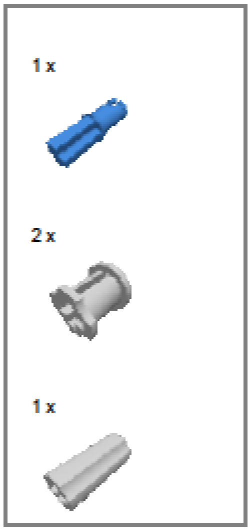

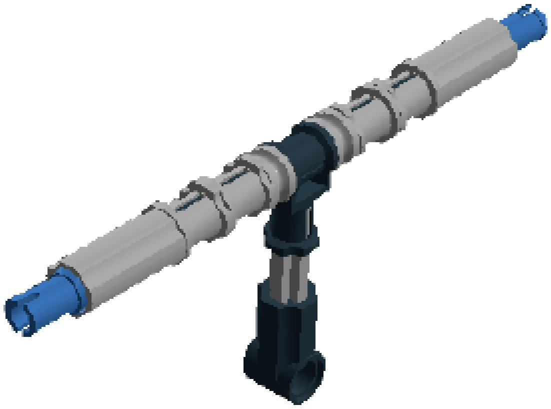

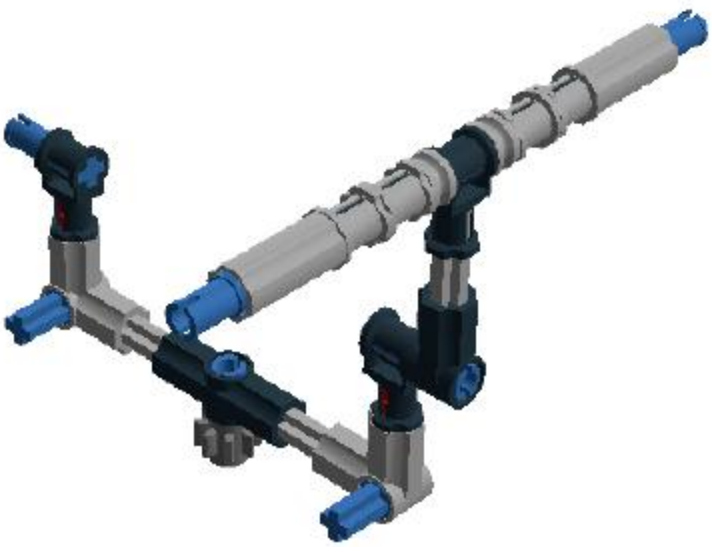

Step 15 of 37

Step 16 of 37

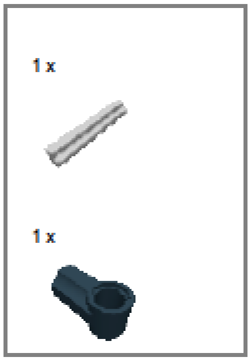

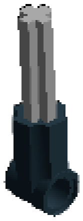

Step 17 of 37

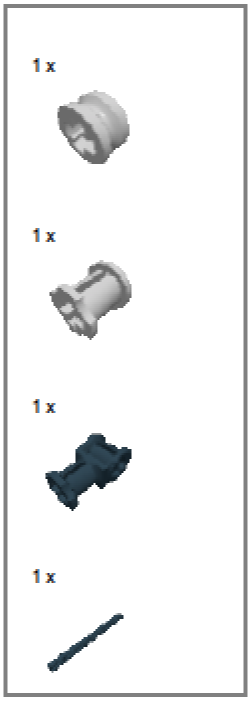

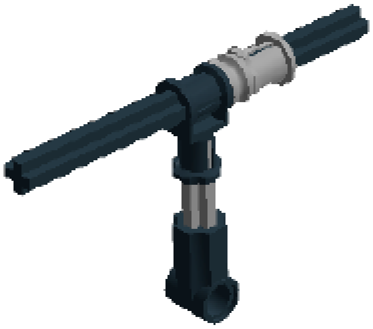

Step 18 of 37

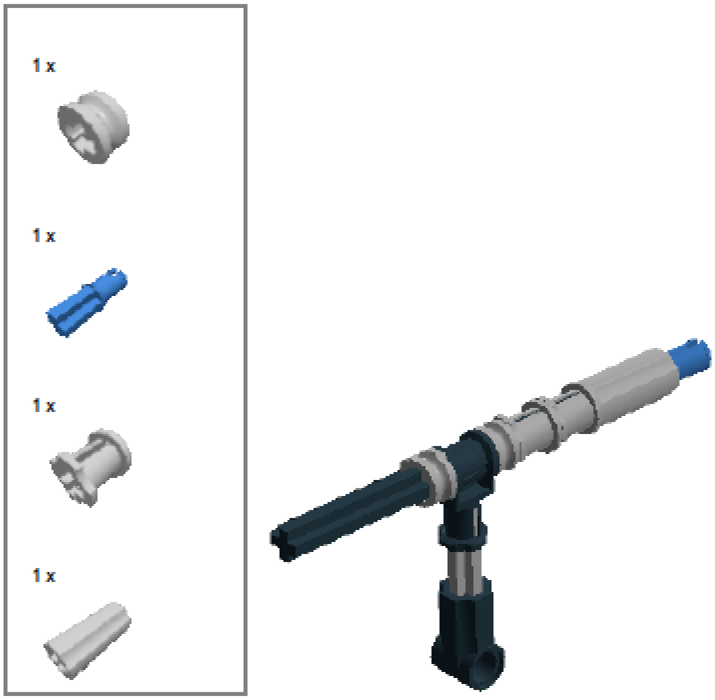

Step 19 of 37

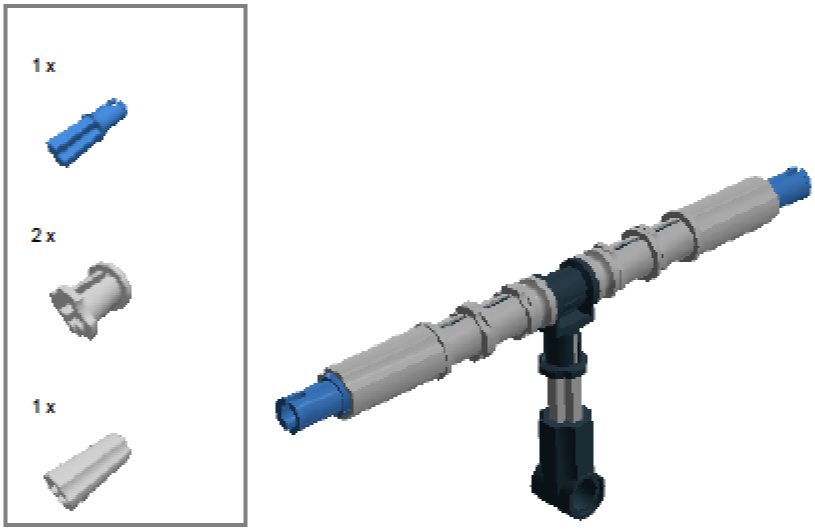

Step 20 of 37

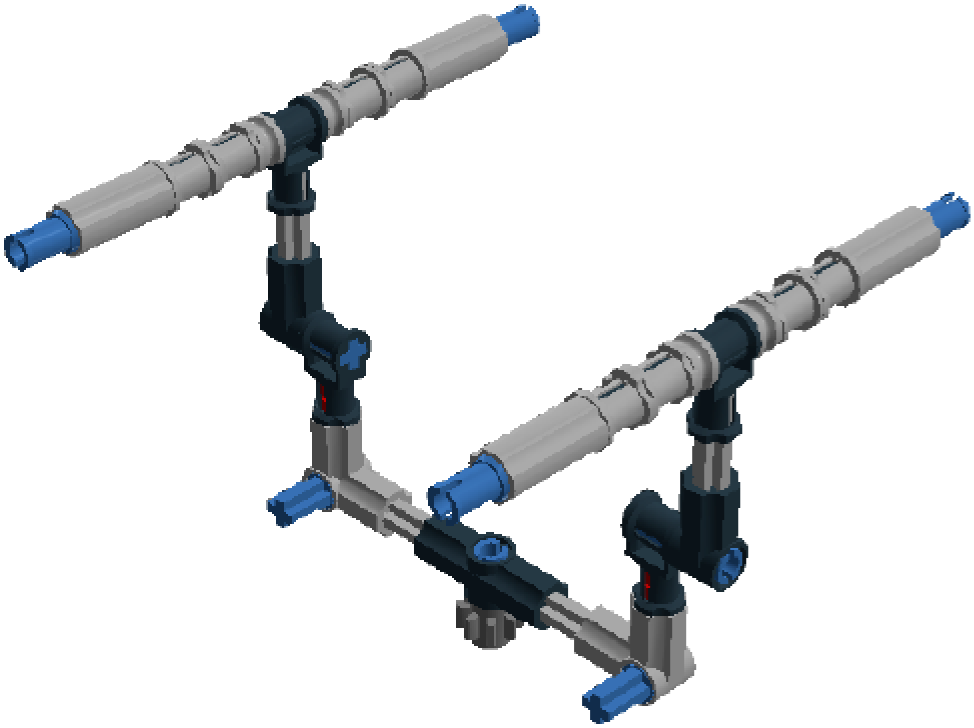

Step 21 of 37

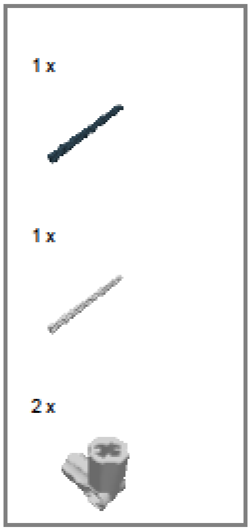

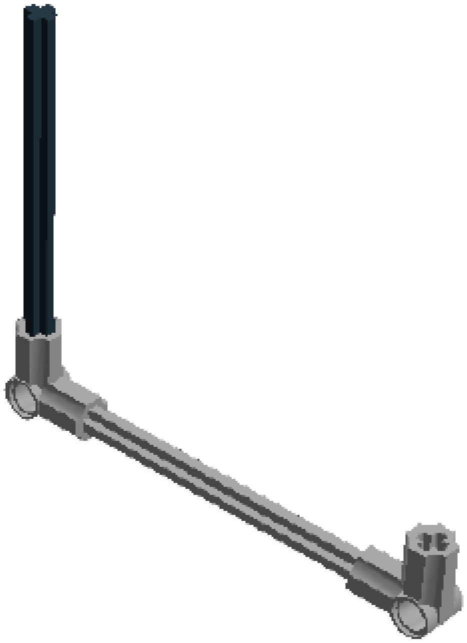

Step 22 of 37

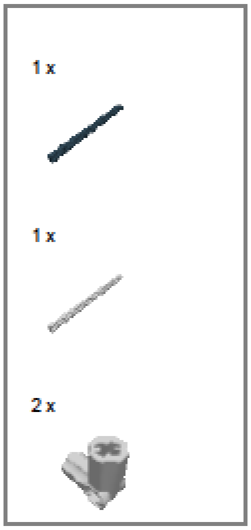

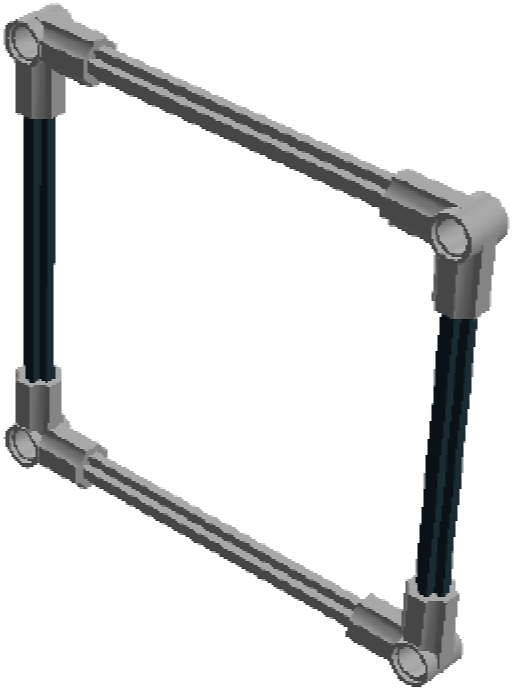

Step 23 of 37

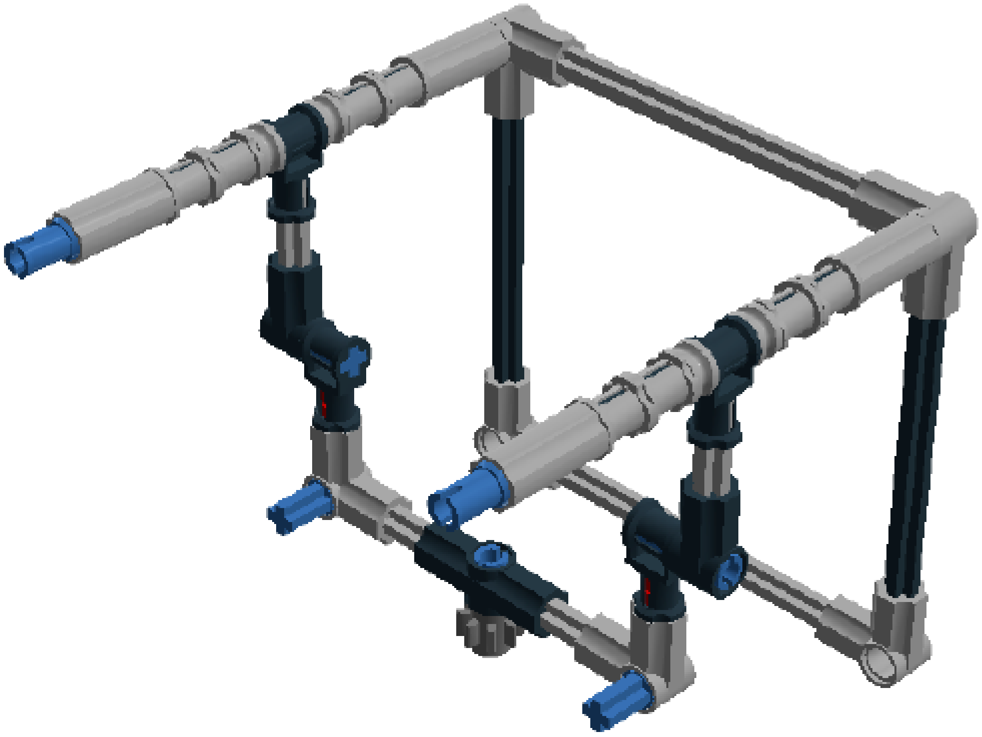

Step 24 of 37

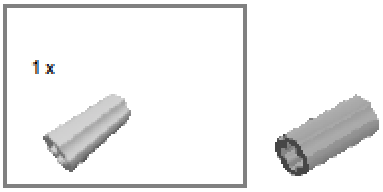

Step 25 of 37

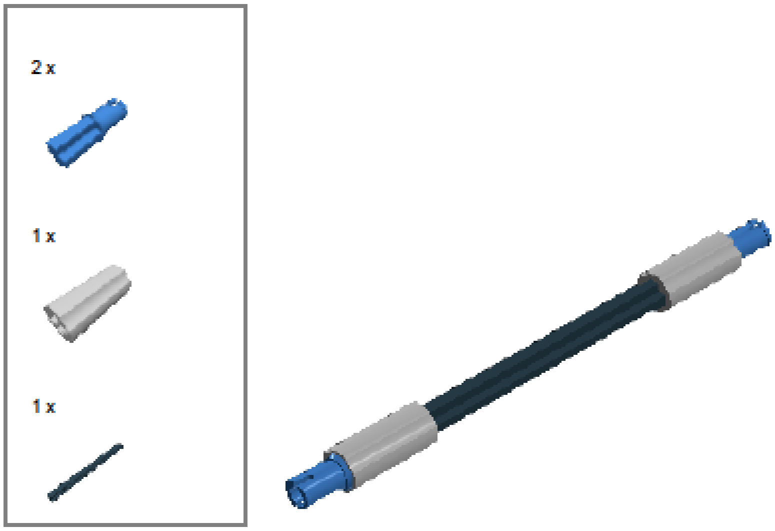

Step 26 of 37

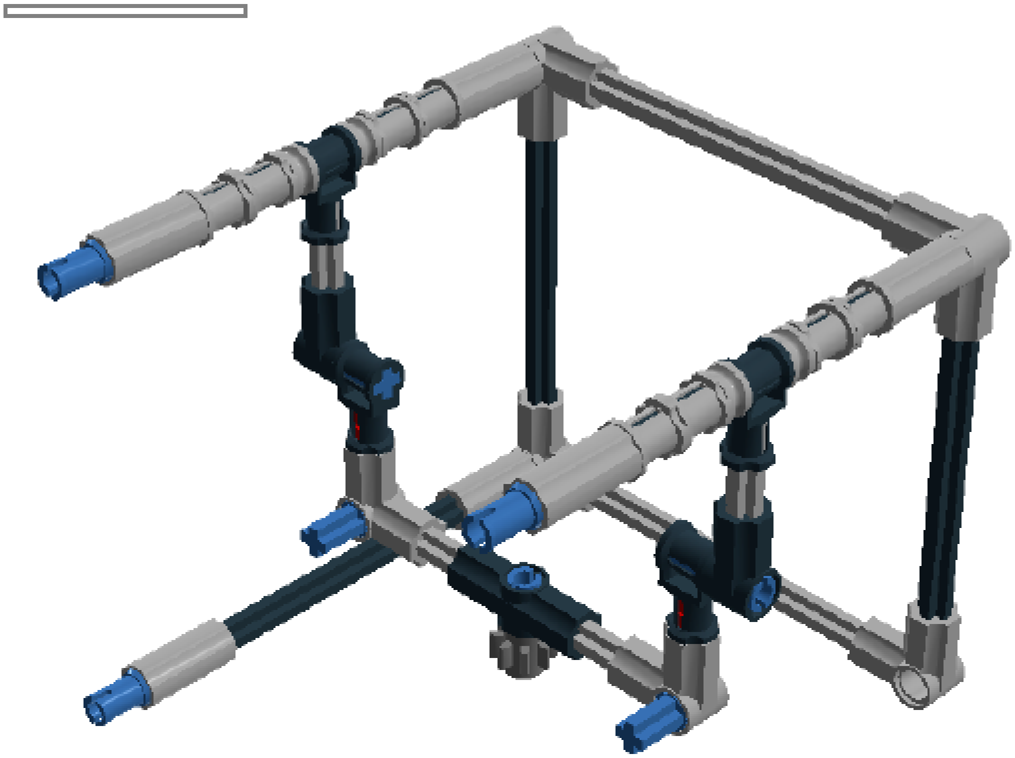

Step 27 of 37

1 x

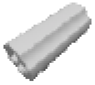

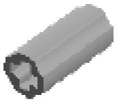

Step 28 of 37

2 x

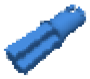

1 x

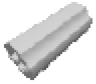

1 x

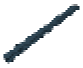

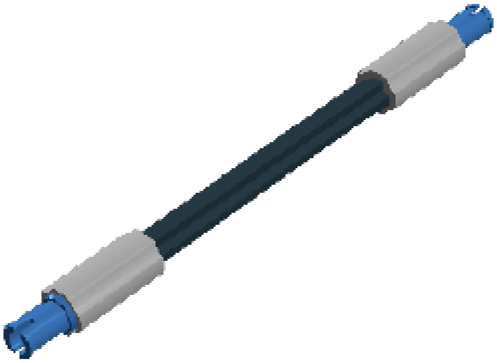

Step 29 of 37

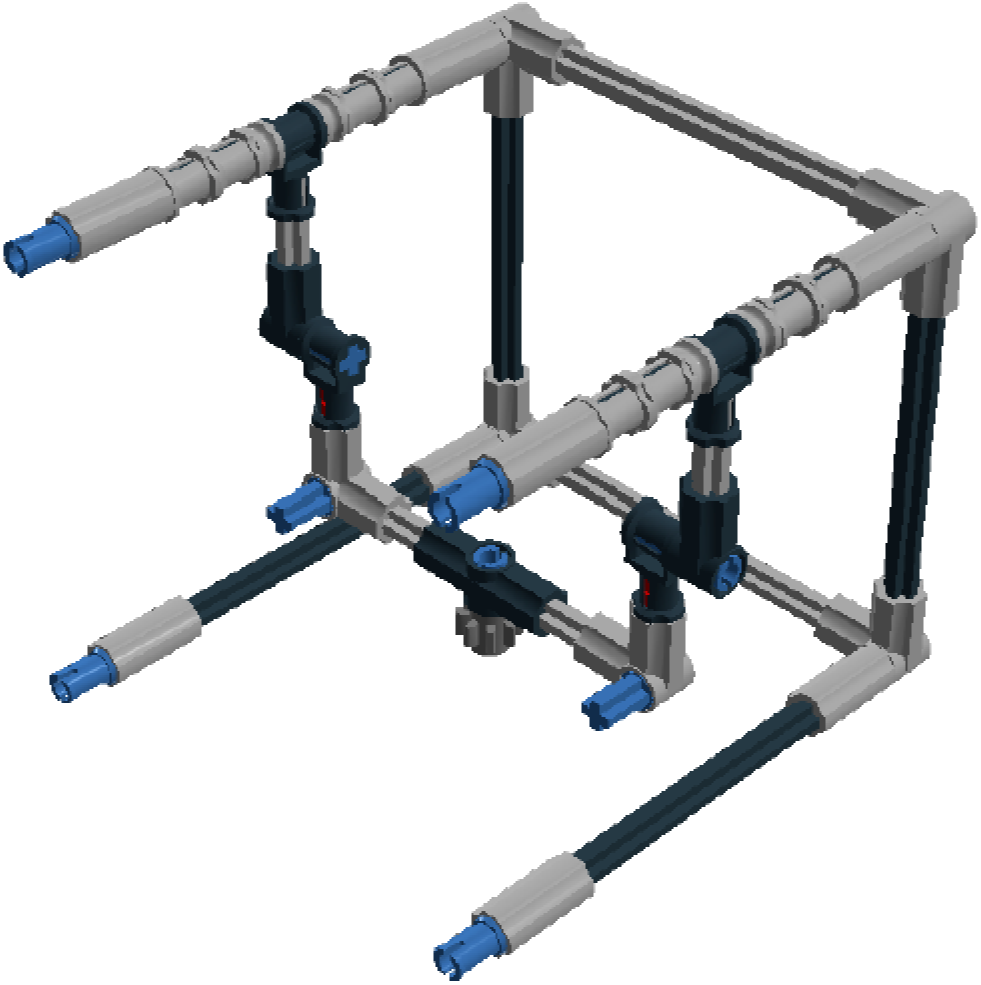

Step 31 of 37

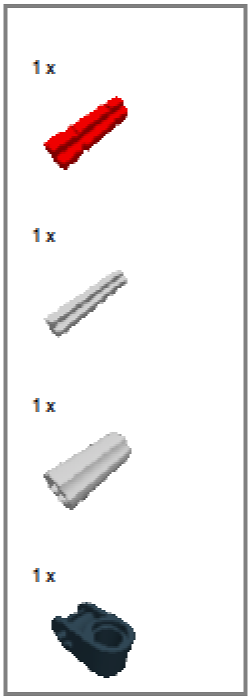

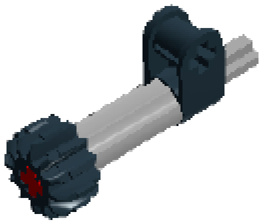

Step 30 of 37

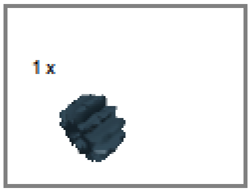

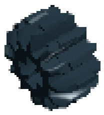

Step 32 of 37

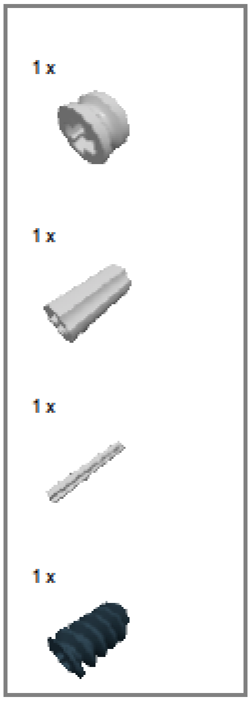

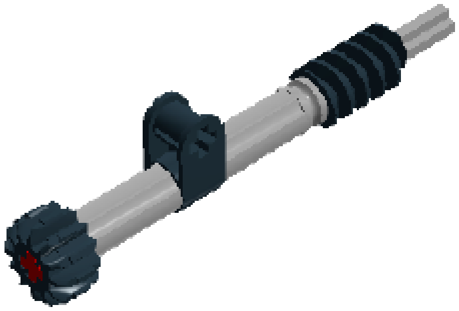

Step 33 of 37

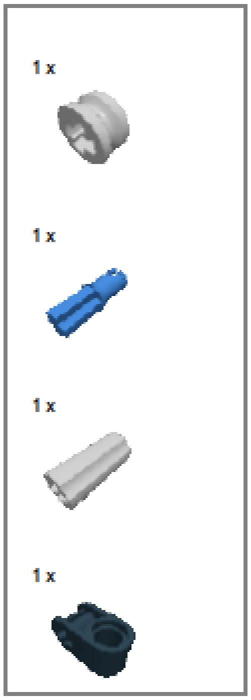

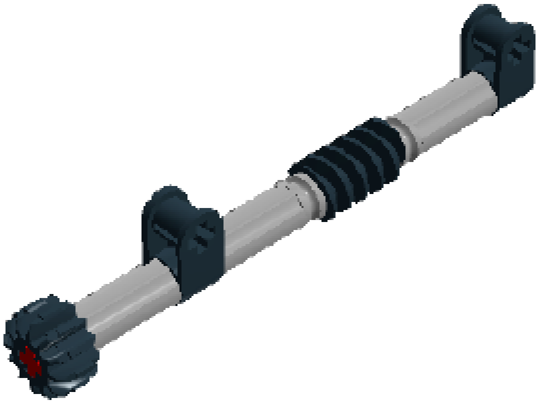

Step 34 of 37

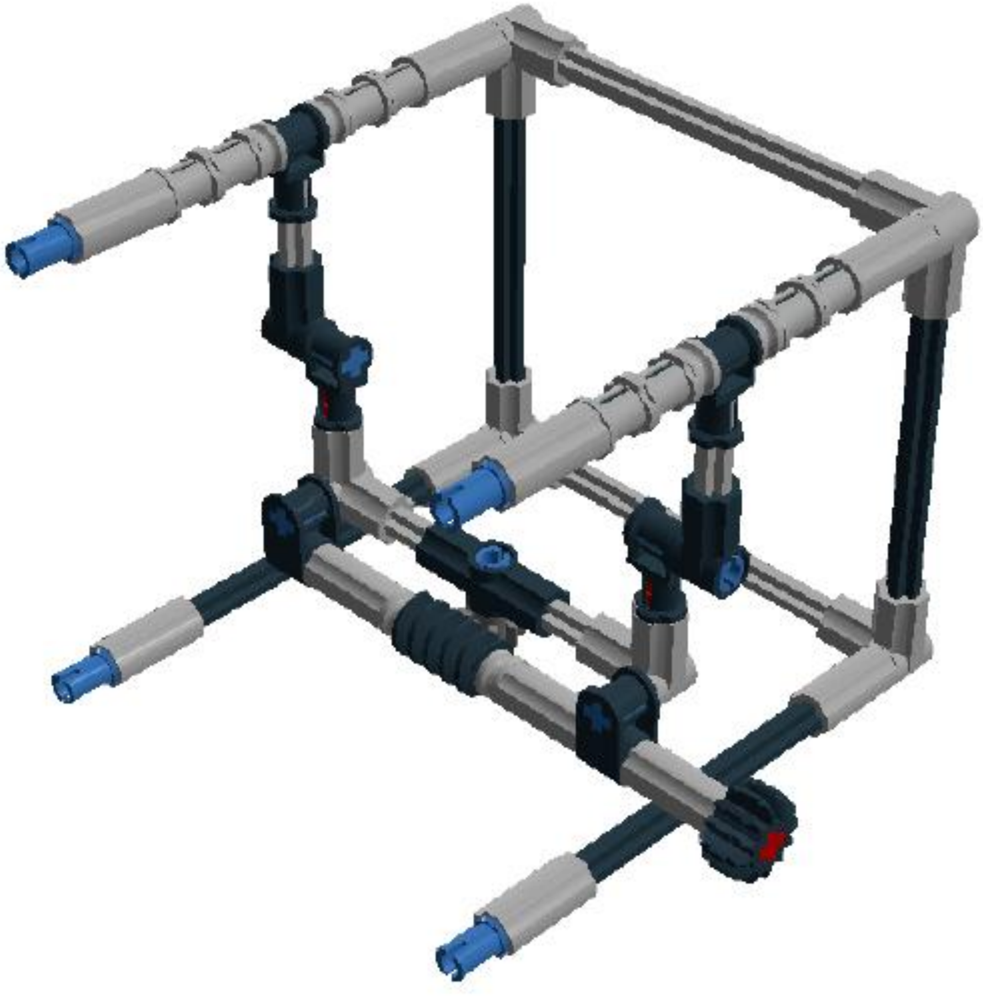

Step 35 of 37

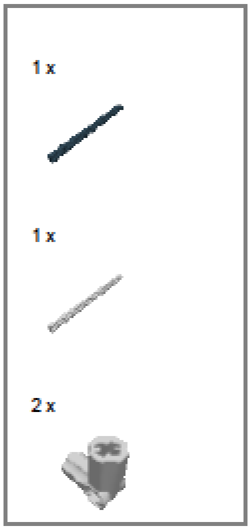

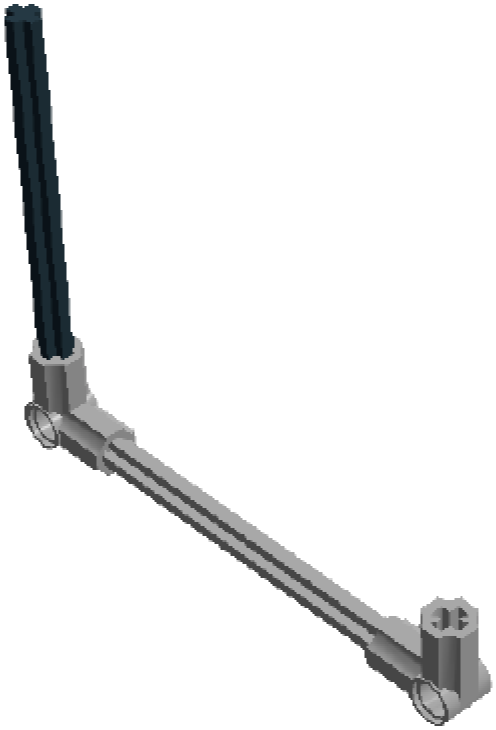

Step 36 of 37

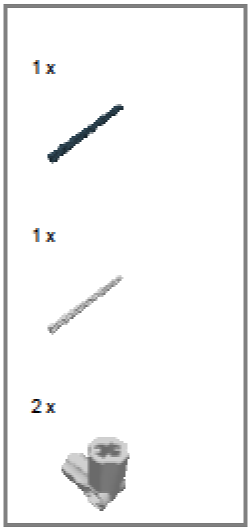

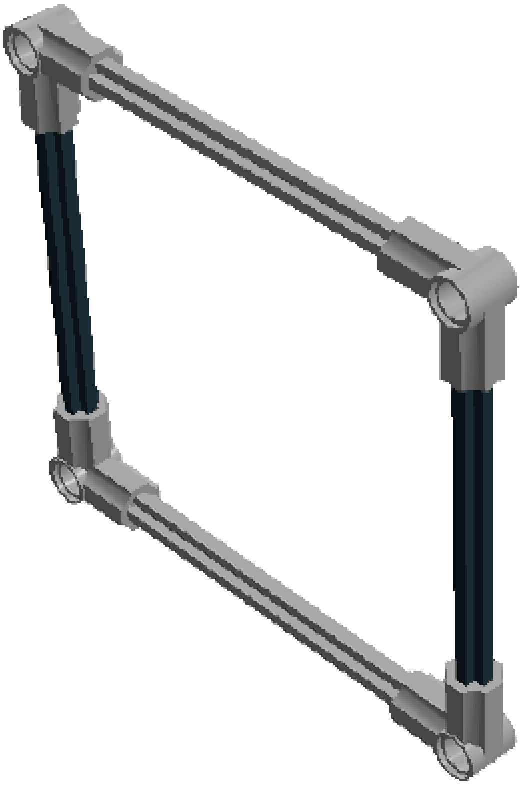

Step 37 of 37

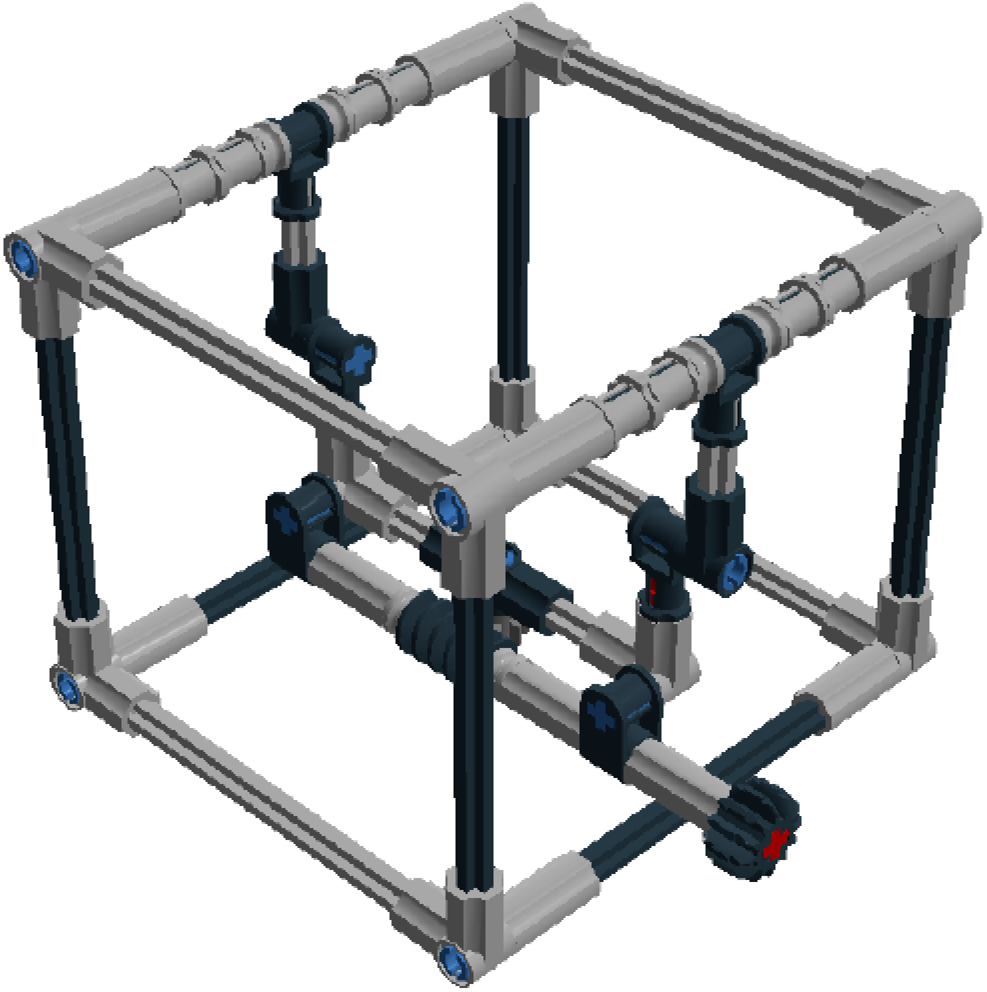

Building instructions[3] for the Open-IMp
Number of bricks = 56

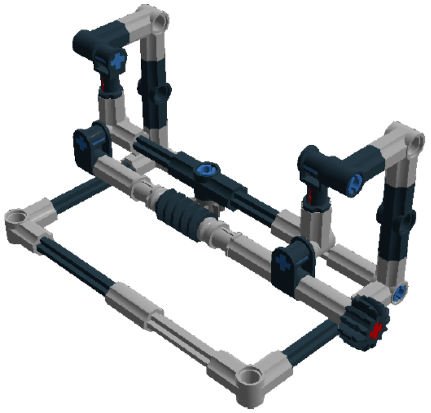

Brick assortment for the Open-Imp
(for correct purchase codes see page 70-73)

Qty	Part no.	Description
2 x	4211573	1/2 BUSH - Medium Stone Grey
9 x	4142865	2M CROSS AXLE W. GROOVE - Bright Red
1 x	4211815	CROSS AXLE 3M - Medium Stone Grey
8 x	4225927	CONNECTOR PEG/CROSS AXLE - Bright Blue
2 x	4211622	BUSH FOR CROSS AXLE - Medium Stone Grey
2 x	370526	CROSS AXLE 4M - Black
6 x	4512360	CROSS AXLE, EXTENSION, 2M - Medium Stone Grey
2 x	4107081	CATCH W. CROSS HOLE - Black
1 x	4211639	CROSS AXLE 5M - Medium Stone Grey
2 x	653626	CROSS BLOCK 90° - Black
2 x	4107085	ANGLE ELEMENT, 0 DEGREES [1] - Black
3 x	370626	CROSS AXLE 6M - Black
1 x	4211805	CROSS AXLE 7M - Medium Stone Grey
3 x	4107783	ANGLE ELEMENT, 180 DEGREES [2] - Black
1 x	4535768	CROSS AXLE 9M - Medium Stone Grey
8 x	4211670	ANGLE ELEMENT, 90 DEGREES [6] - Medium Stone Grey
1 x	4514559	GEAR WHEEL T=8, M=1 - Dark Stone Grey
1 x	4177431	DOUBLE CONICAL WHEEL Z12 1M - Black
1 x	471626	WORM - Black

3 As generated by LEGO® digital designer 4.3.8

Step 1 of 27

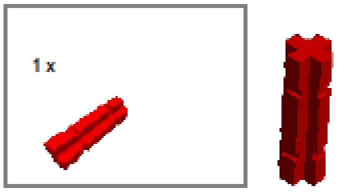

Step 2 of 27

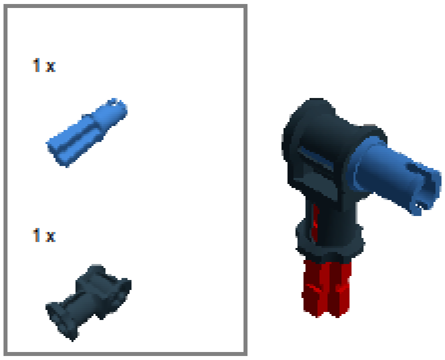

Step 3 of 27

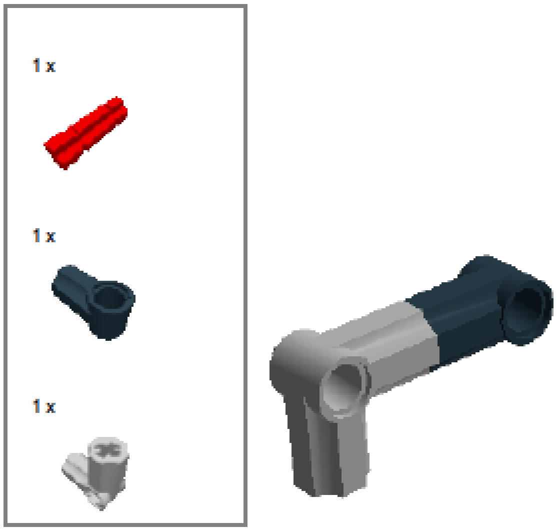

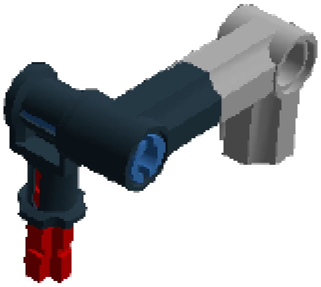

Step 4 of 27

Step 5 of 27

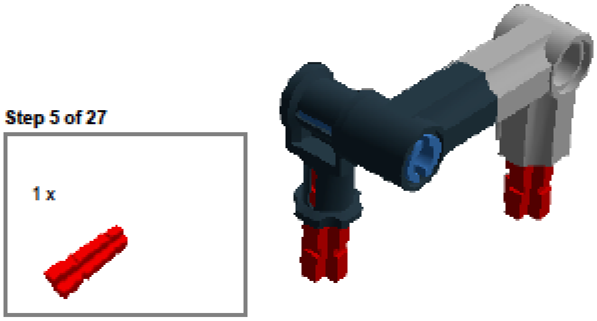

Step 6 of 27

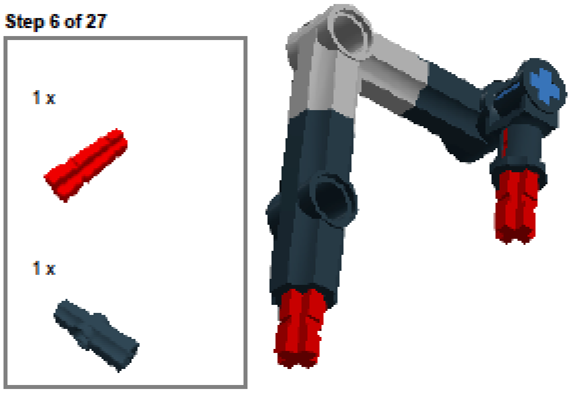

Step 7 of 27

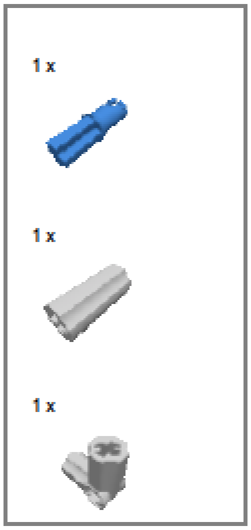

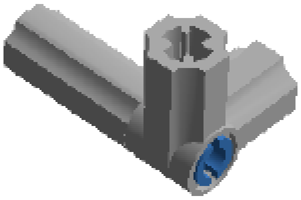

Step 8 of 27

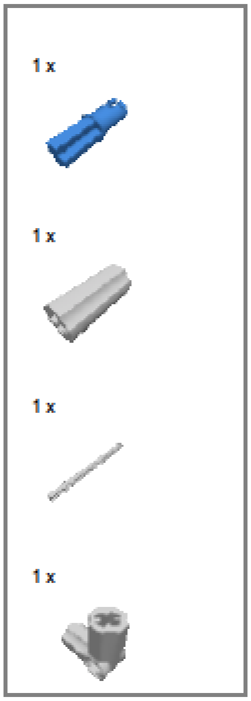

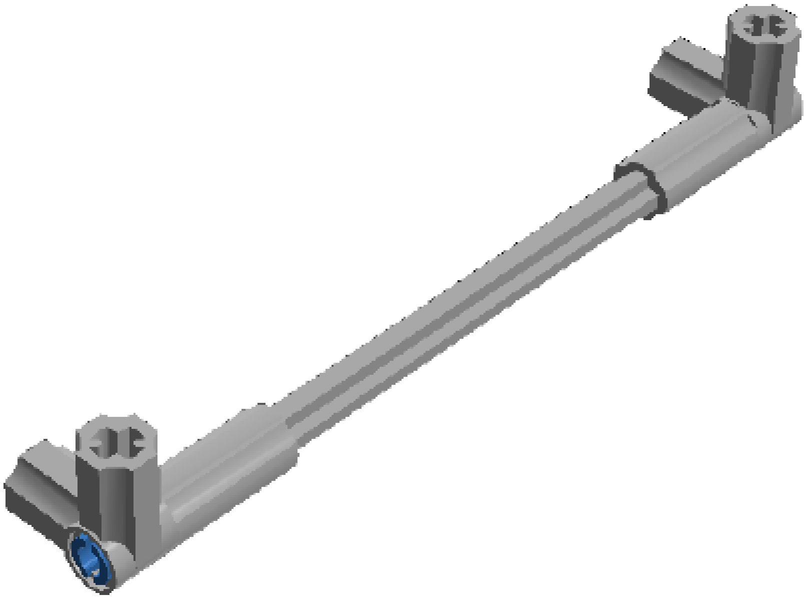

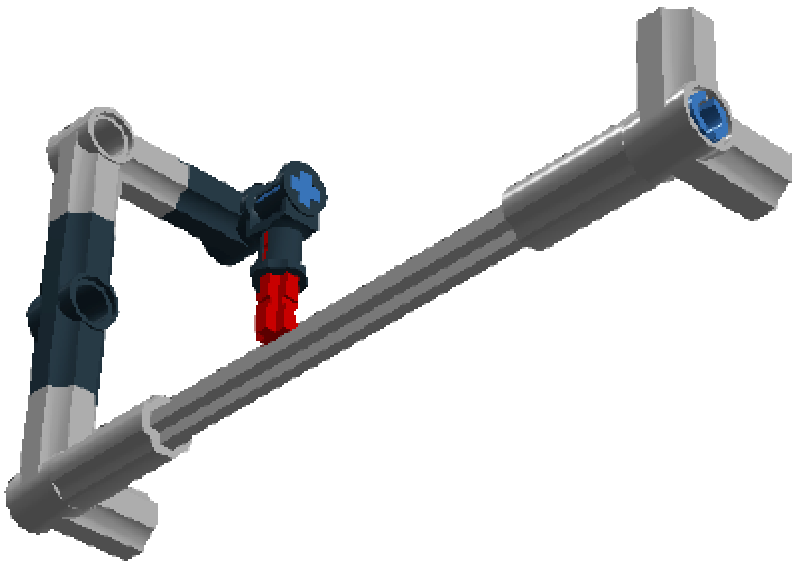

Step 9 of 27

Step 10 of 27

2 x

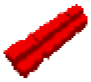

1 x

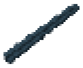

1 x

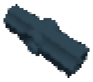

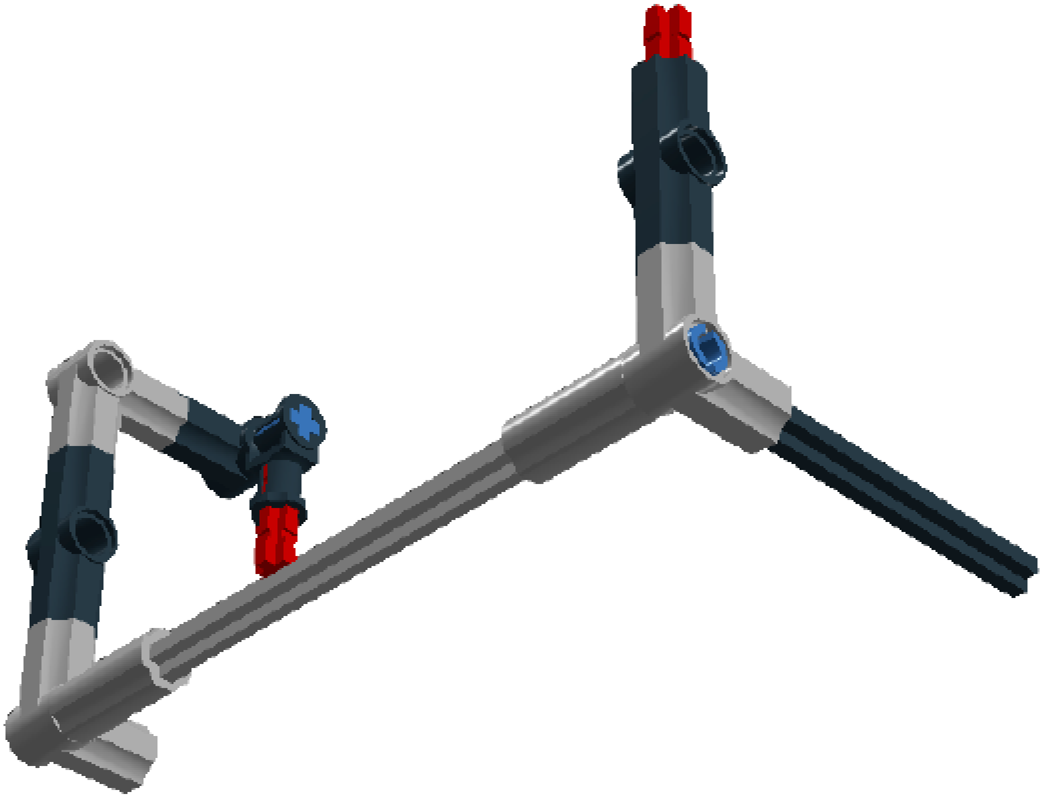

Step 11 of 27

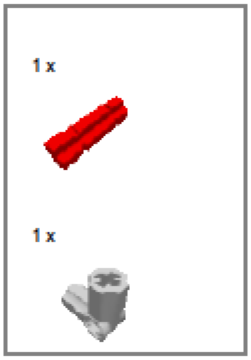

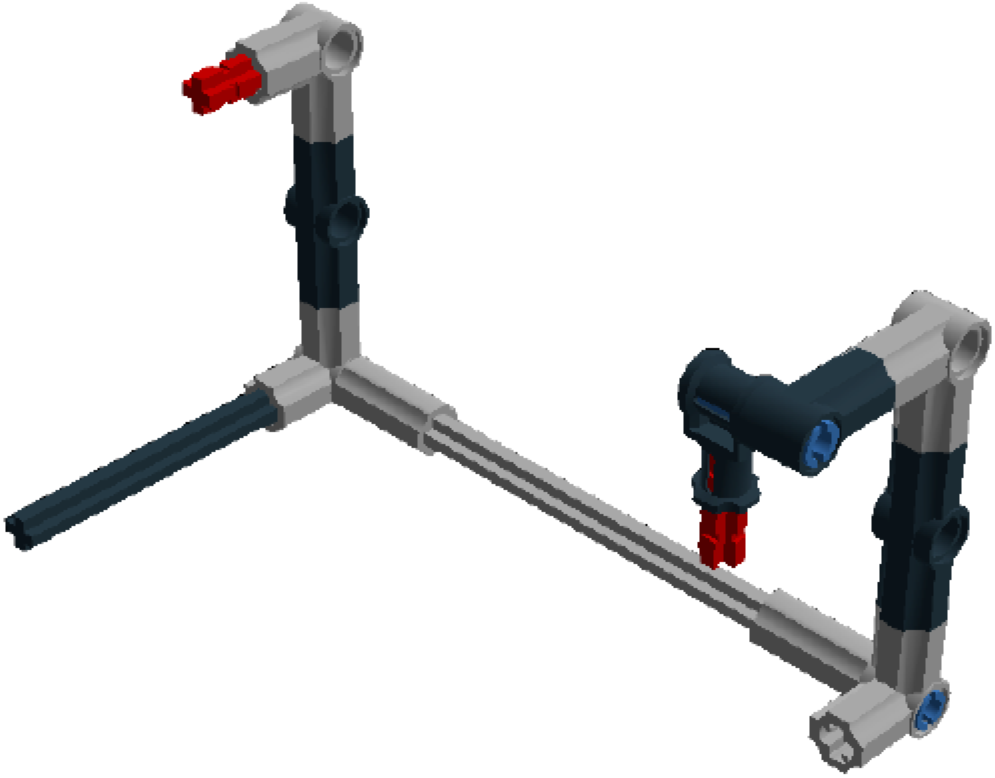

Step 12 of 27

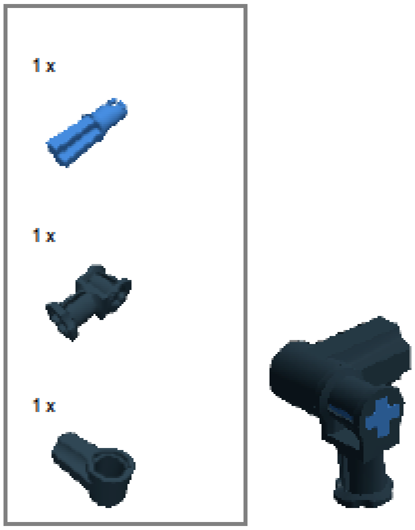

Step 13 of 27

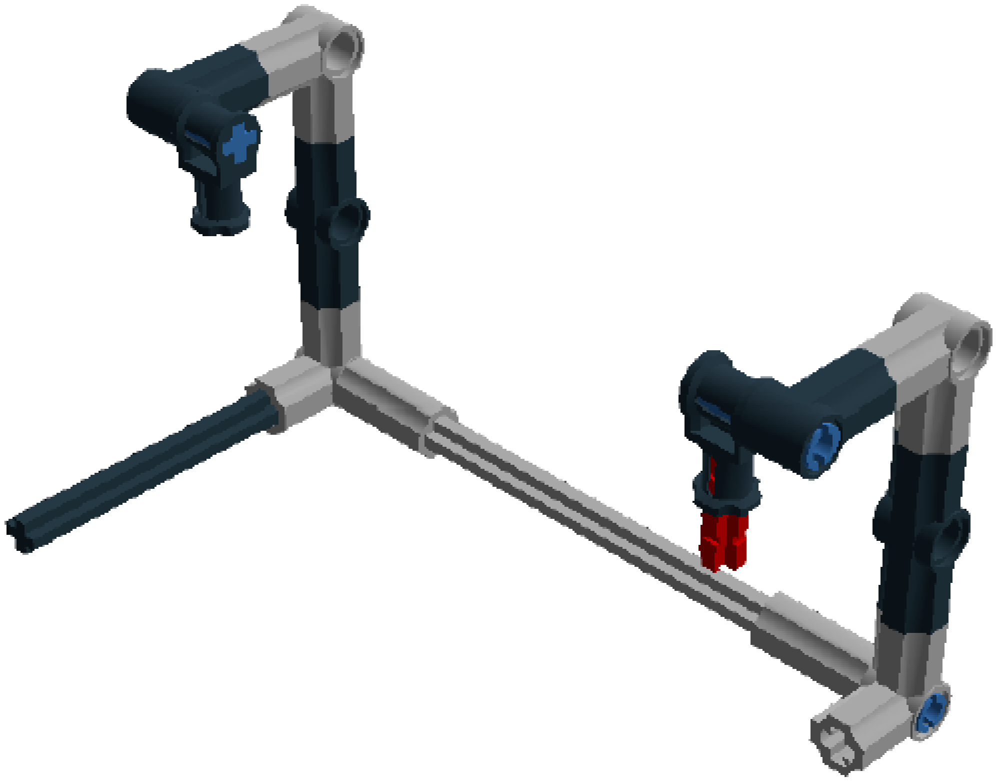

Step 14 of 27

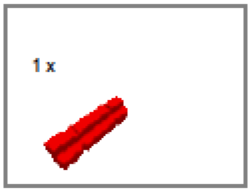

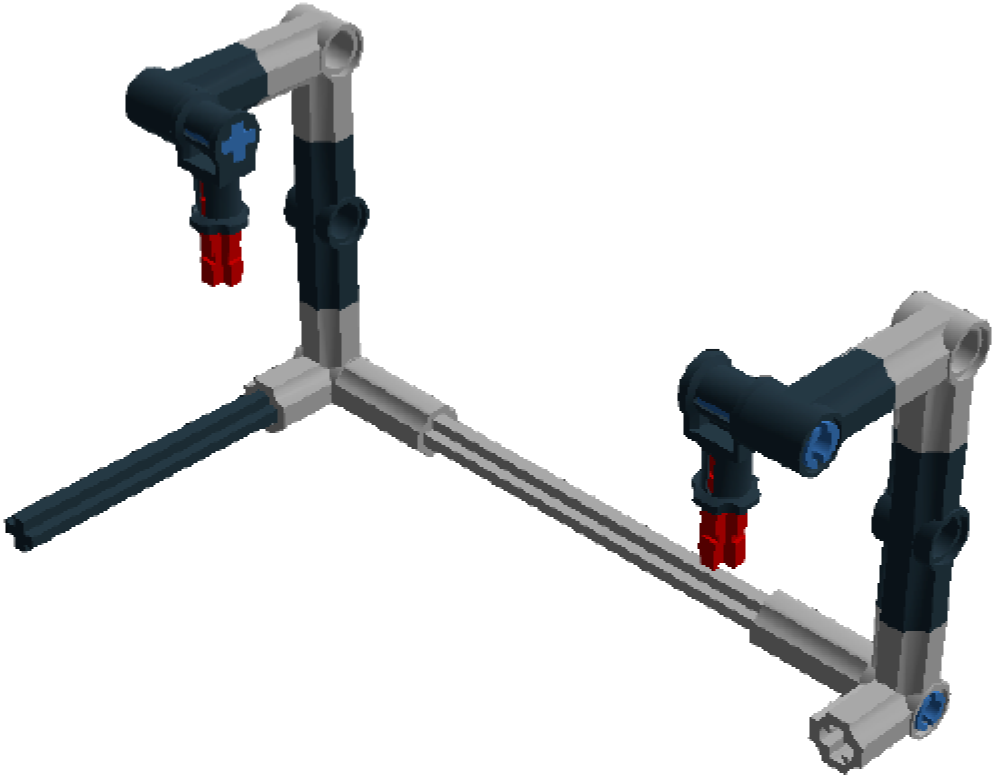

Step 15 of 27

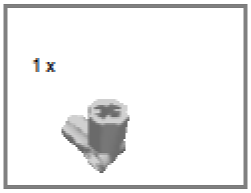

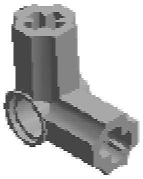

Step 16 of 27

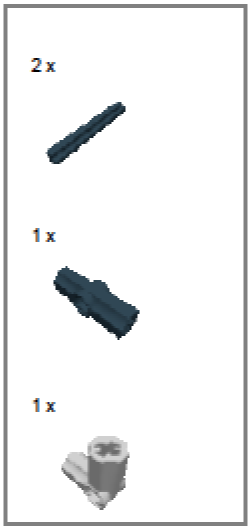

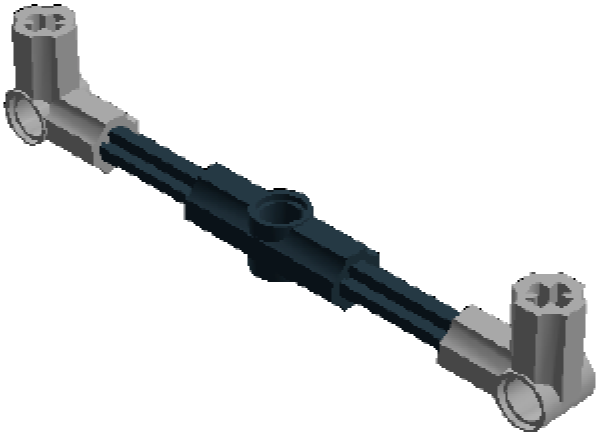

Step 17 of 27

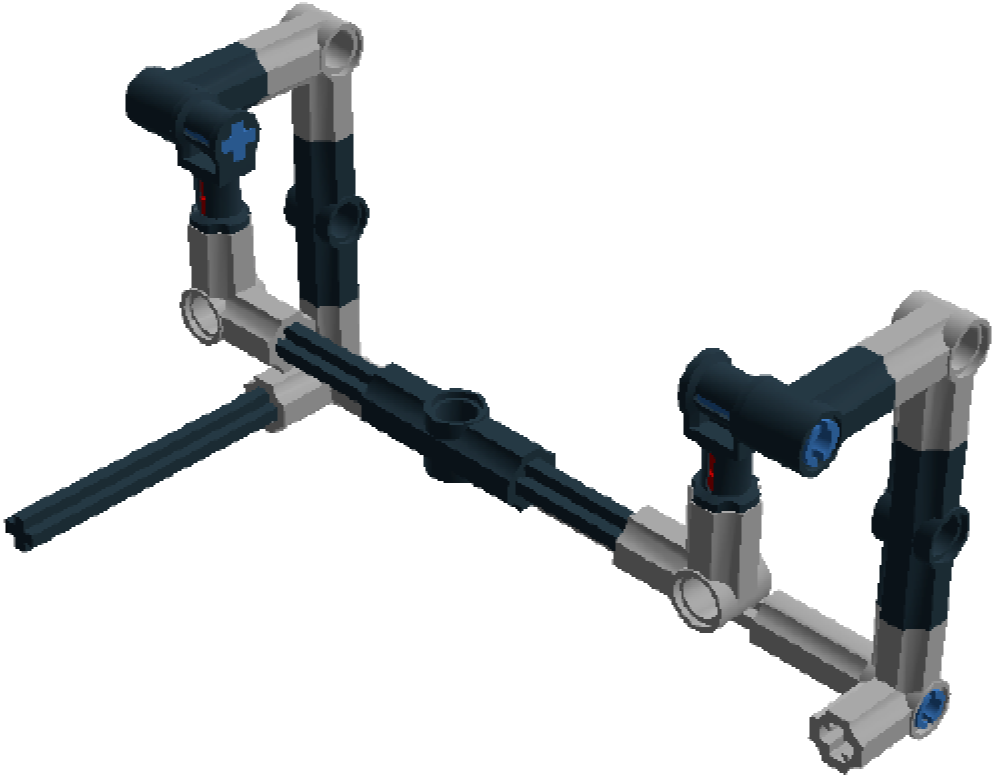

Step 18 of 27

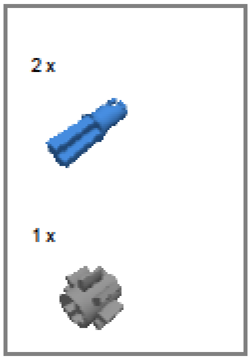

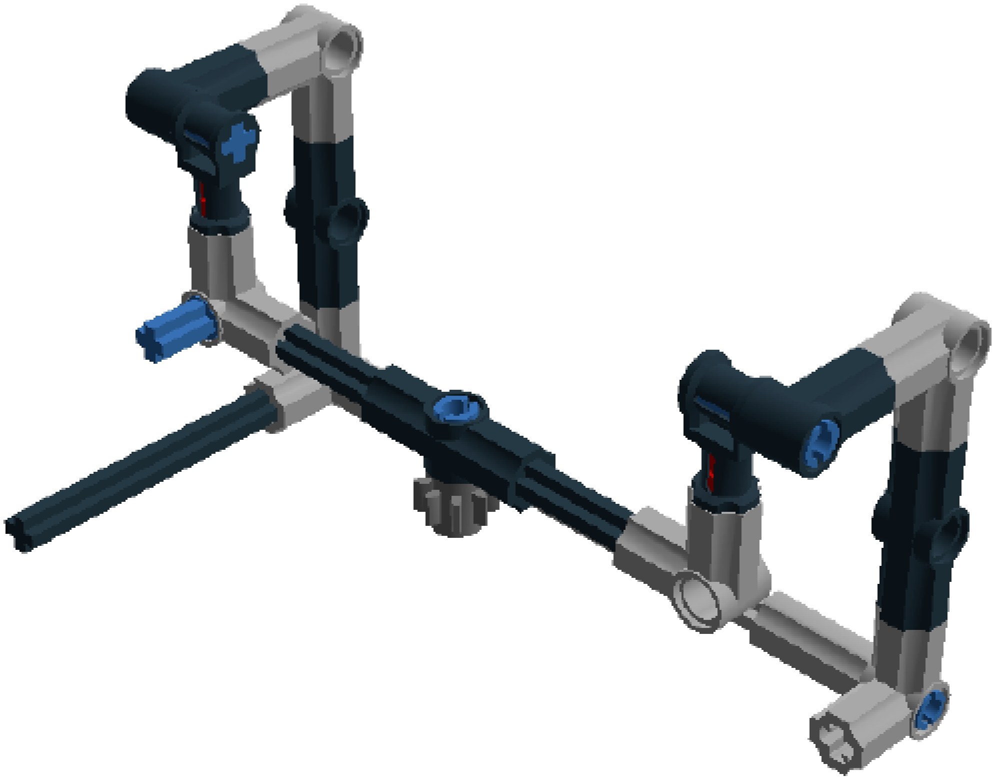

Step 19 of 27

1 x

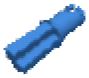

1 x

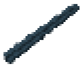

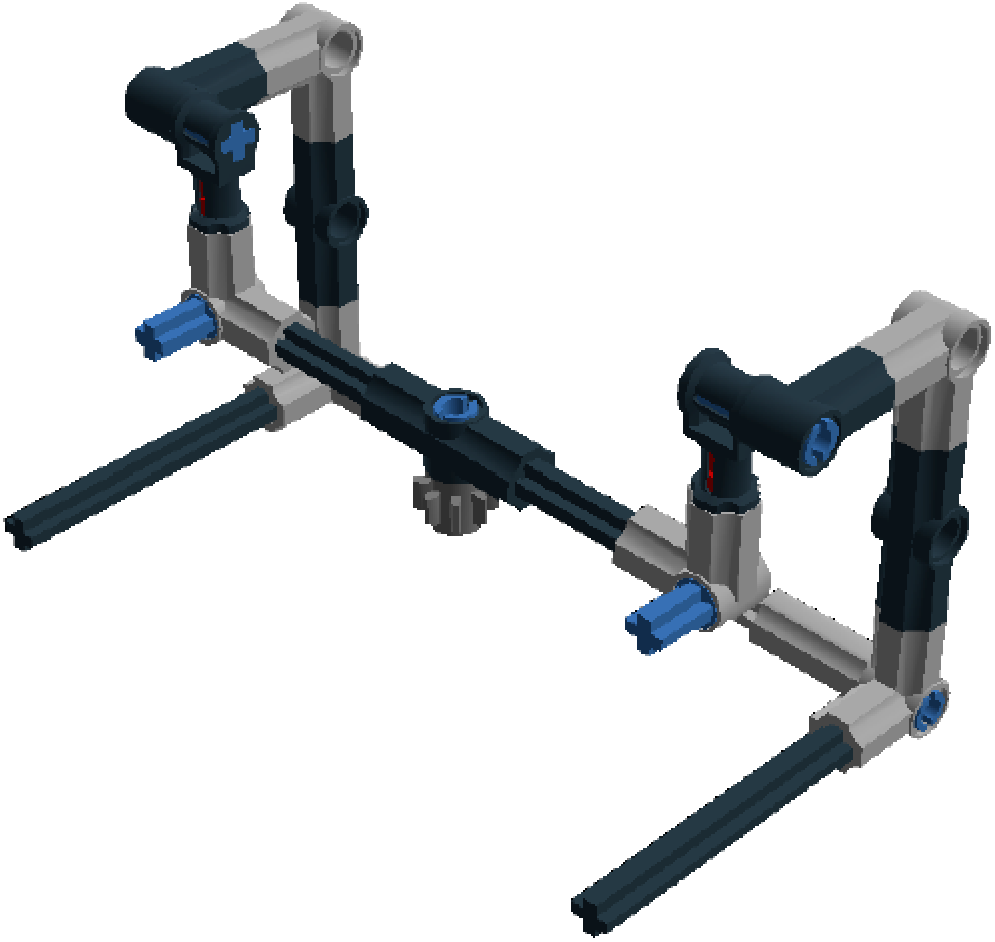

Step 20 of 27

1 x

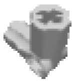

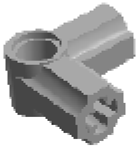

Step 21 of 27

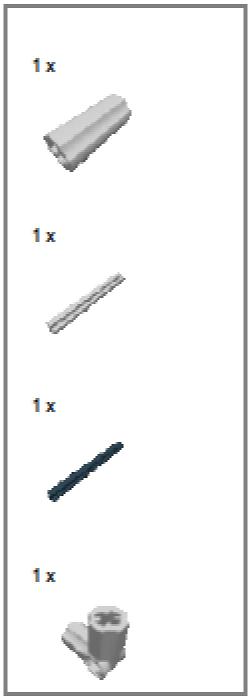

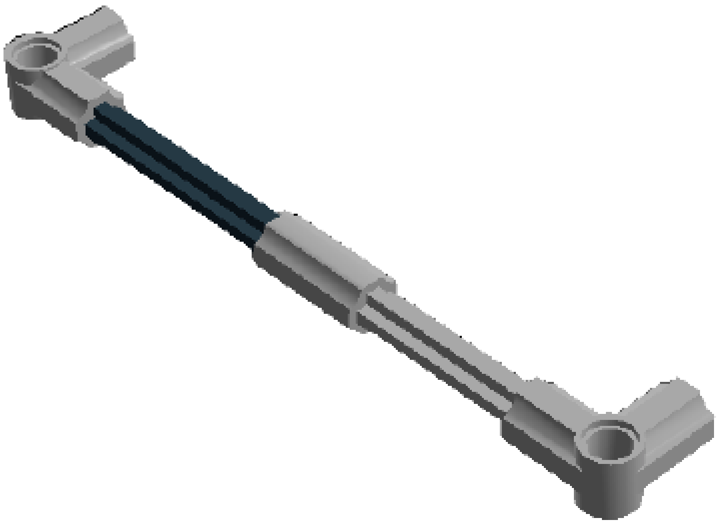

Step 22 of 27

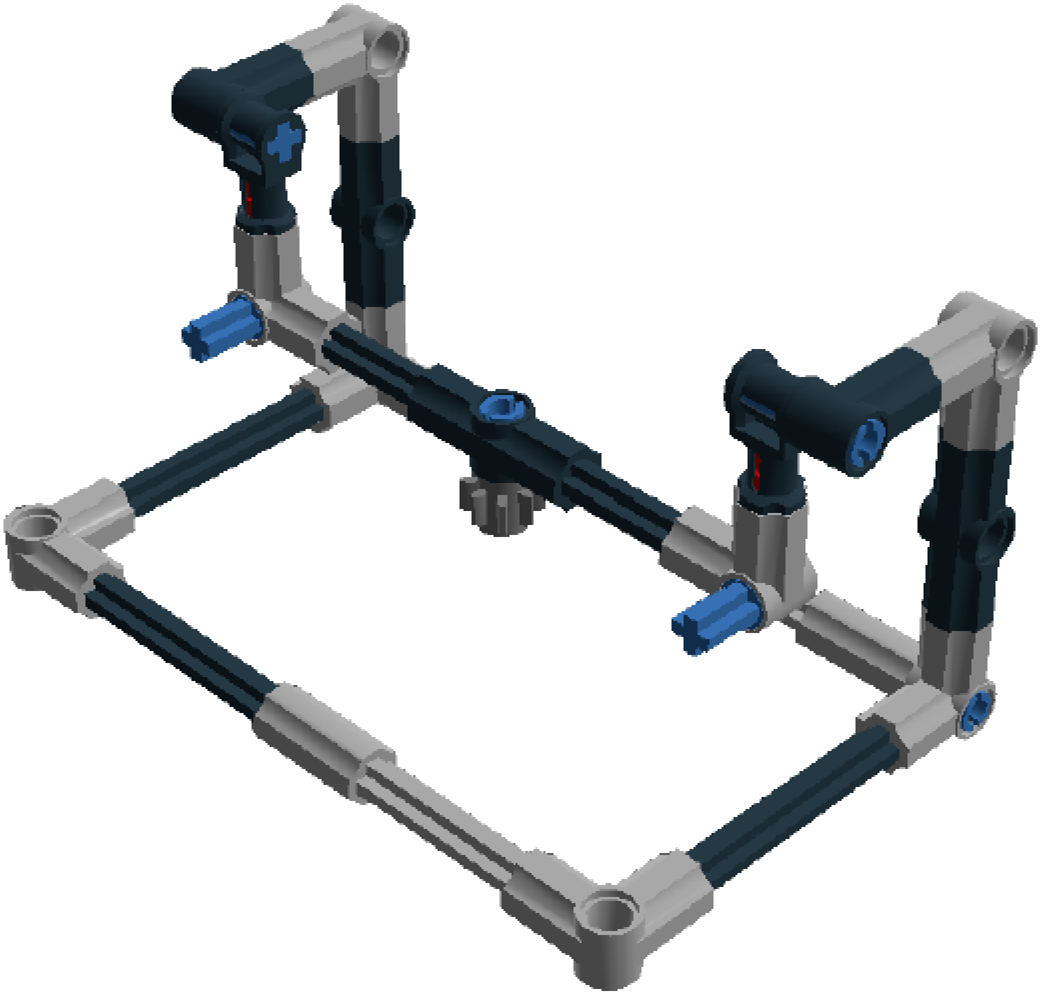

Step 23 of 27

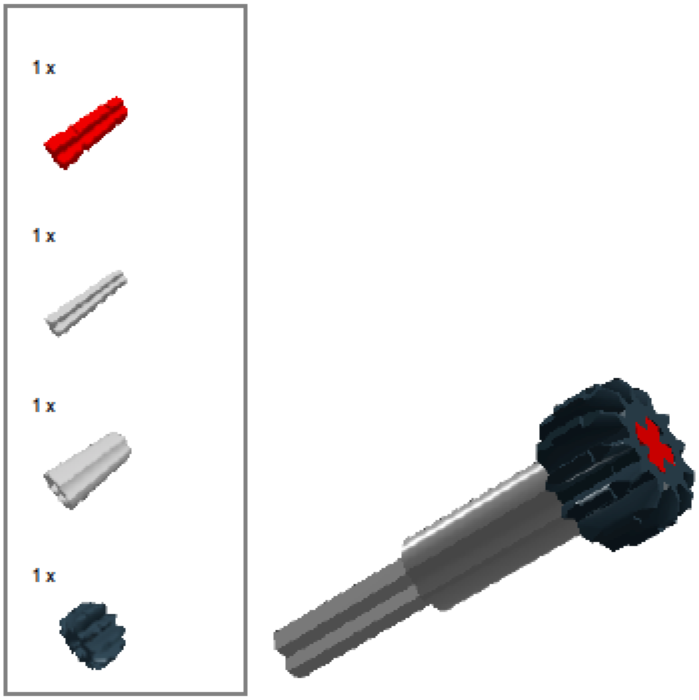

Step 24 of 27

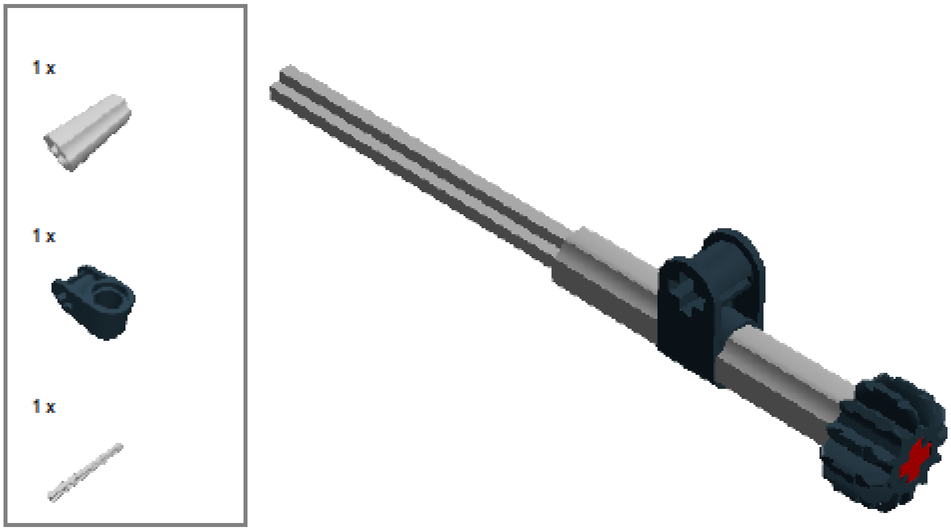

Step 25 of 27

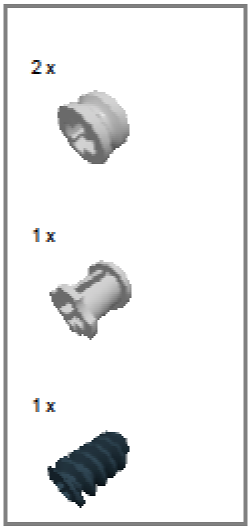

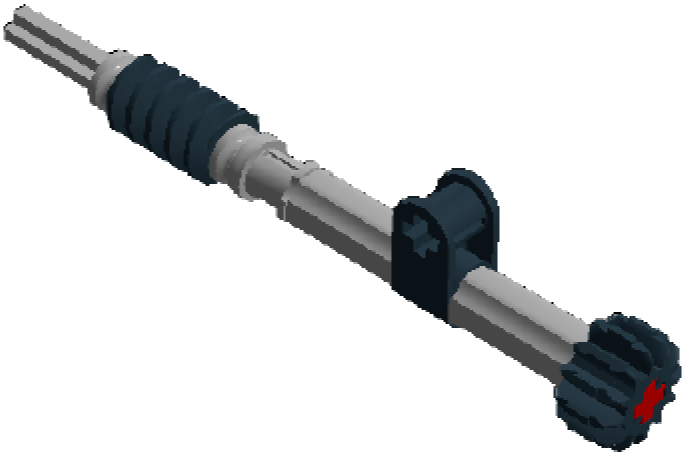

Step 26 of 27

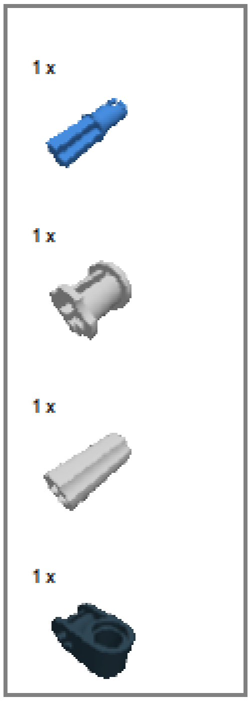

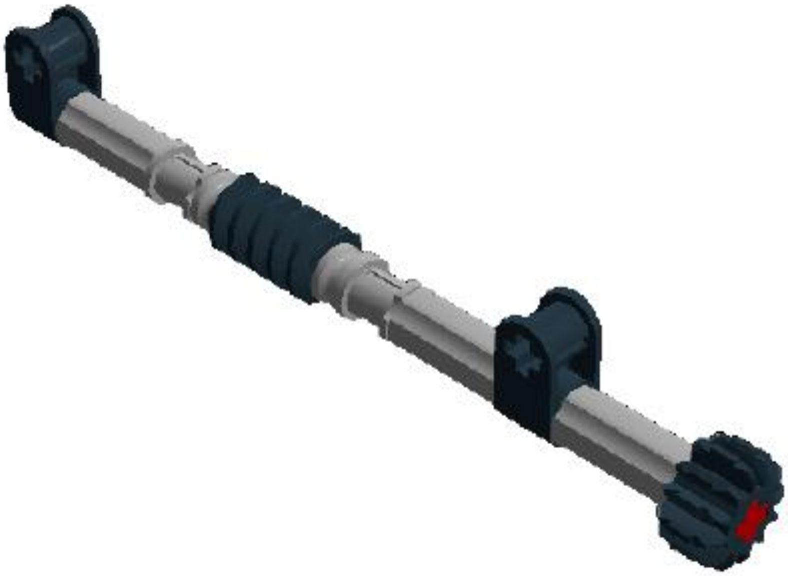

Step 27 of 27

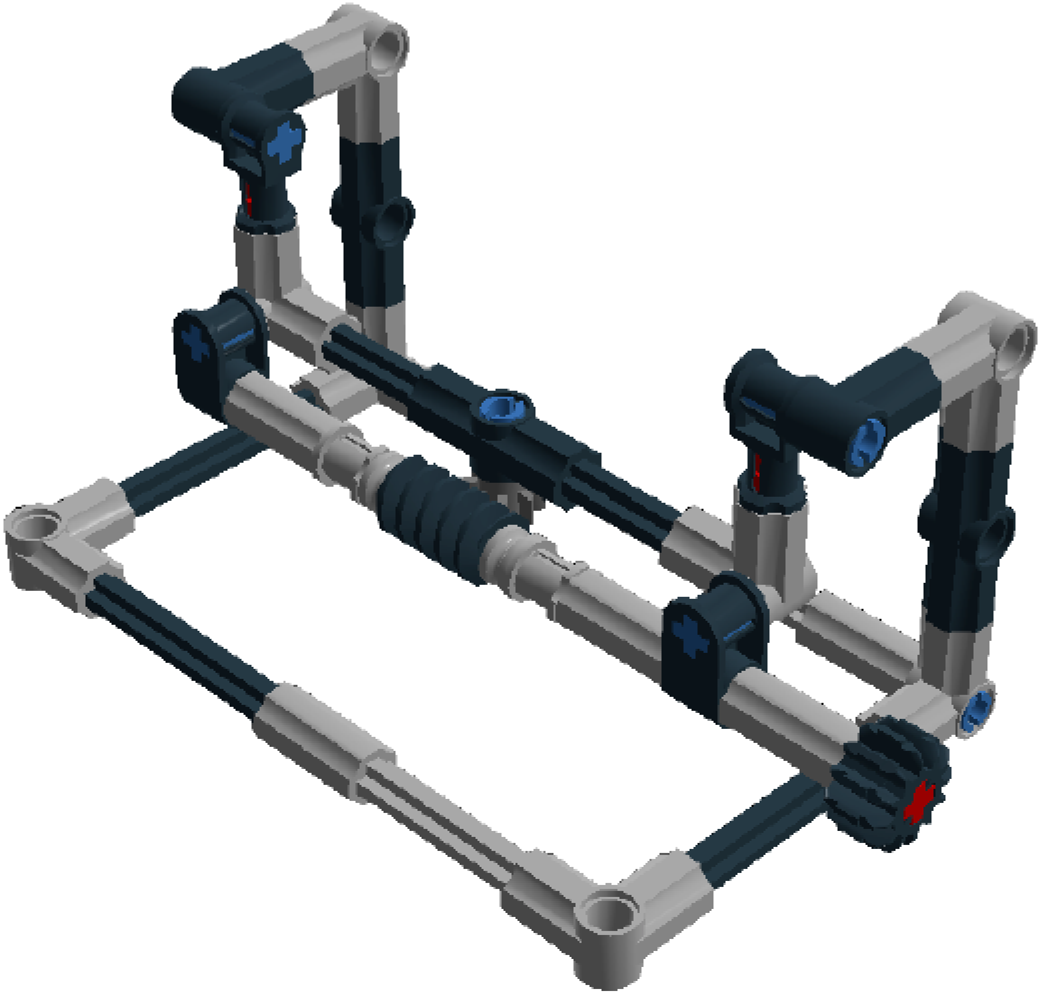

Building instructions[4] for the Giant-IMp
Number of bricks = 132

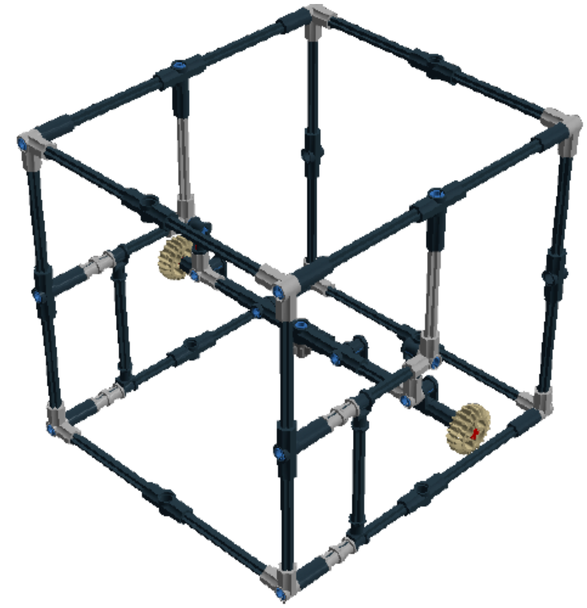

Brick assortment for the Giant-Imp
(for correct purchase codes see page 70-73)

2 x 4211573 1/2 BUSH - Medium Stone Grey	8 x 4142865 2M CROSS AXLE W. GROOVE - Bright Red	2 x 4211815 CROSS AXLE 3M - Medium Stone Grey
18 x 4225927 CONNECTOR PEG/CROSS AXLE - Bright Blue	10 x 4211622 BUSH FOR CROSS AXLE - Medium Stone Grey	2 x 370526 CROSS AXLE 4M - Black
18 x 4512363 CROSS AXLE, EXTENSION, 2M - Black	6 x 4107081 CATCH W. CROSS HOLE - Black	4 x 653626 CROSS BLOCK 90° - Black
2 x 4211805 CROSS AXLE 7M - Medium Stone Grey	28 x 370726 CROSS AXLE 8M - Black	15 x 4107783 ANGLE ELEMENT, 180 DEGREES [2] - Black
1 x 4535768 CROSS AXLE 9M - Medium Stone Grey	12 x 4211670 ANGLE ELEMENT, 90 DEGREES [6] - Medium Stone Grey	1 x 4514559 GEAR WHEEL T=8, M=1 - Dark Stone Grey
1 x 471626 WORM - Black	2 x 4514555 DOUBLE CONICAL WHEEL Z20 1M - Brick Yellow	

[4] As generated by LEGO® digital designer 4.3.8

Step 1 of 48

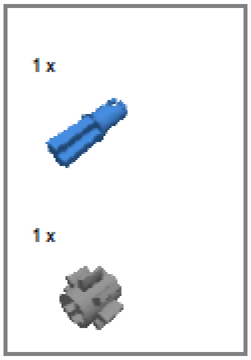

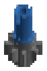

Step 2 of 48

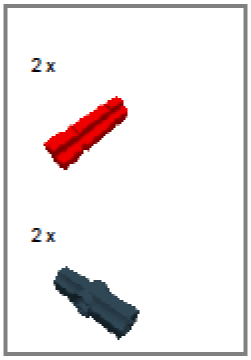

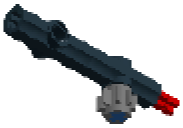

Step 3 of 48

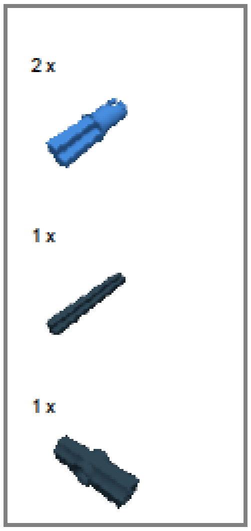

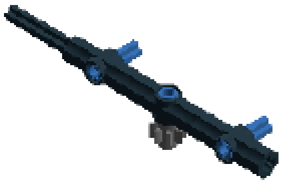

Step 4 of 48

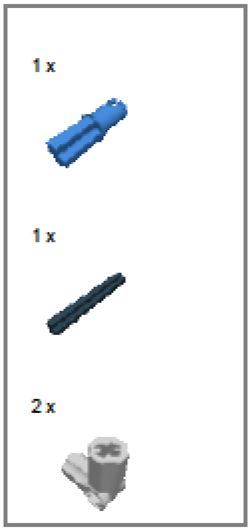

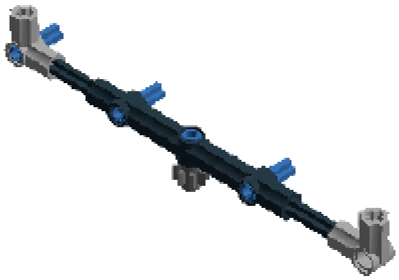

Step 5 of 48

2 x

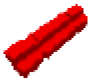

1 x

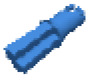

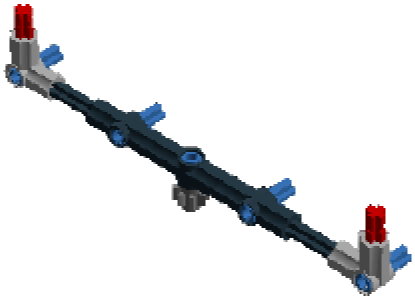

Step 6 of 48

1 x

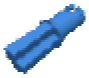

1 x

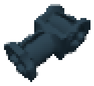

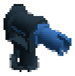

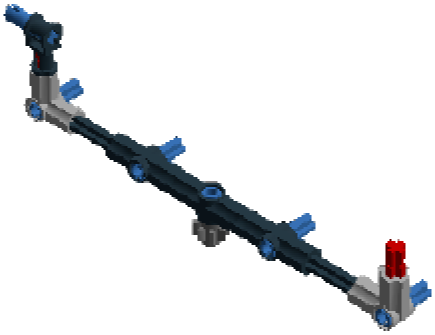

Step 7 of 48

Step 8 of 48

1 x

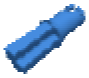

1 x

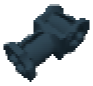

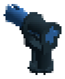

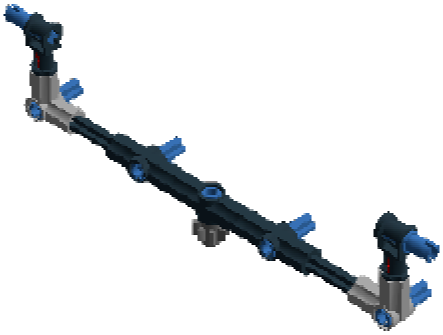

Step 9 of 48

Step 10 of 48

1 x

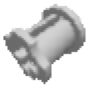

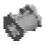

Step 11 of 48

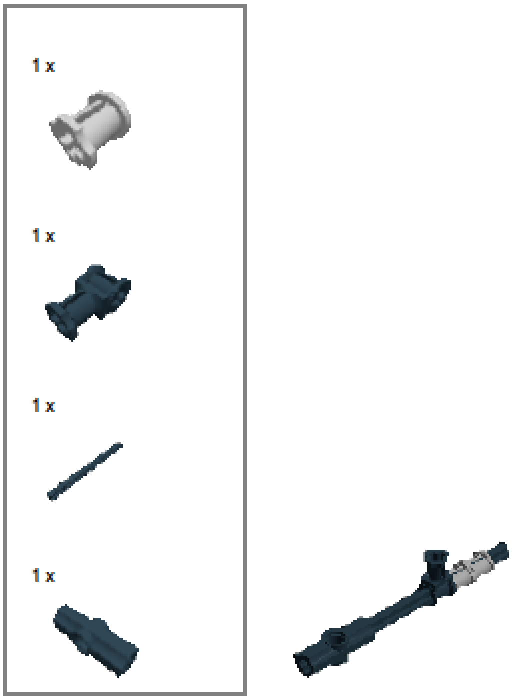

Step 12 of 48

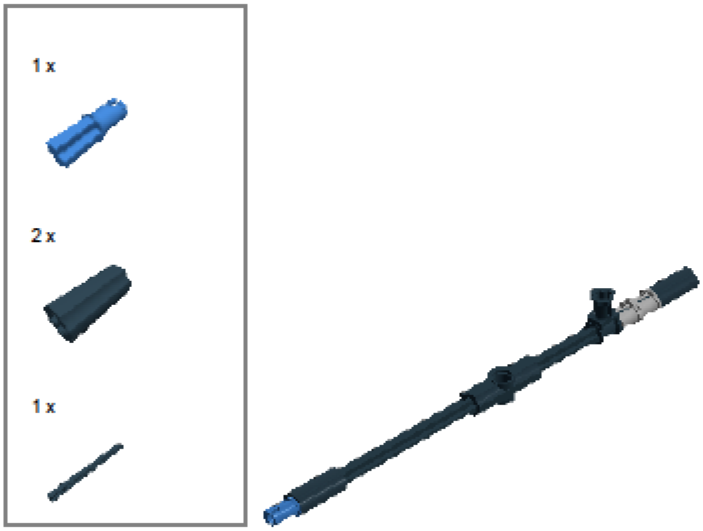

Step 13 of 48

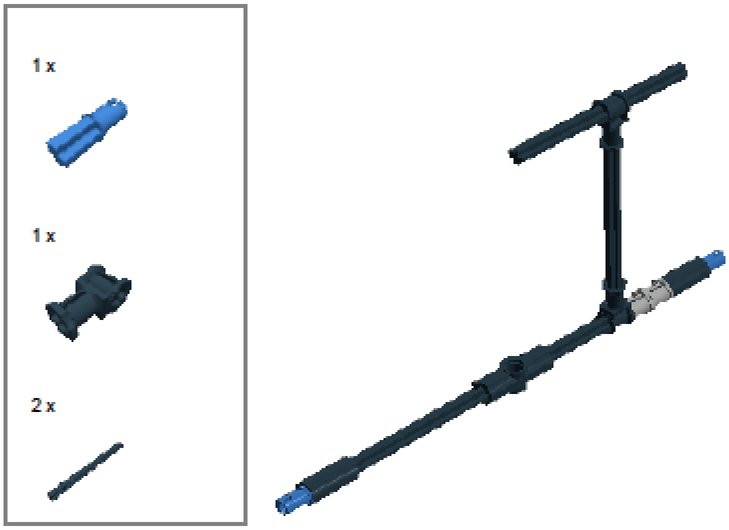

Step 14 of 48

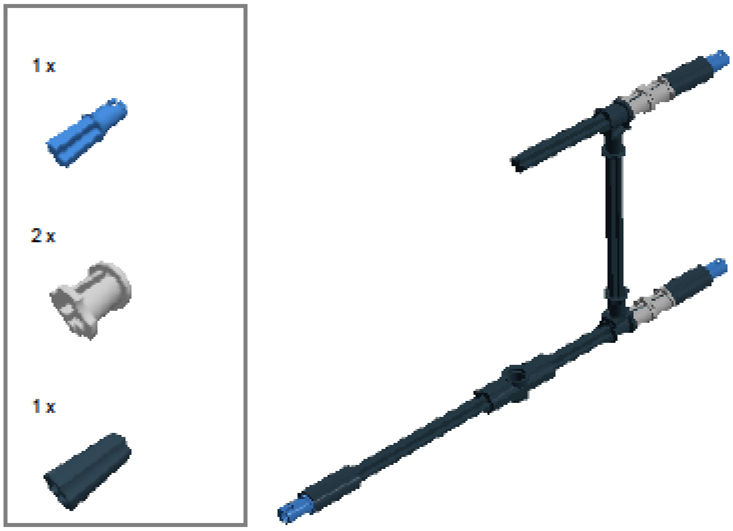

Step 15 of 48

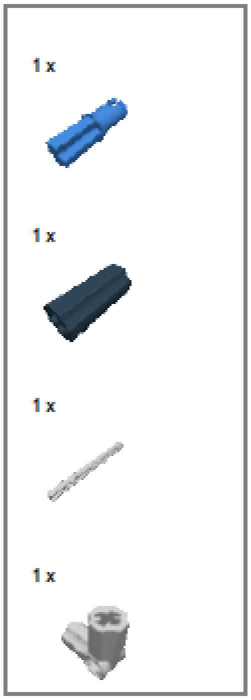

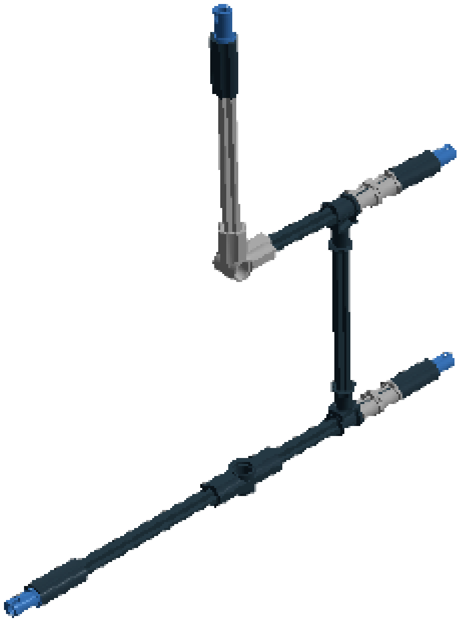

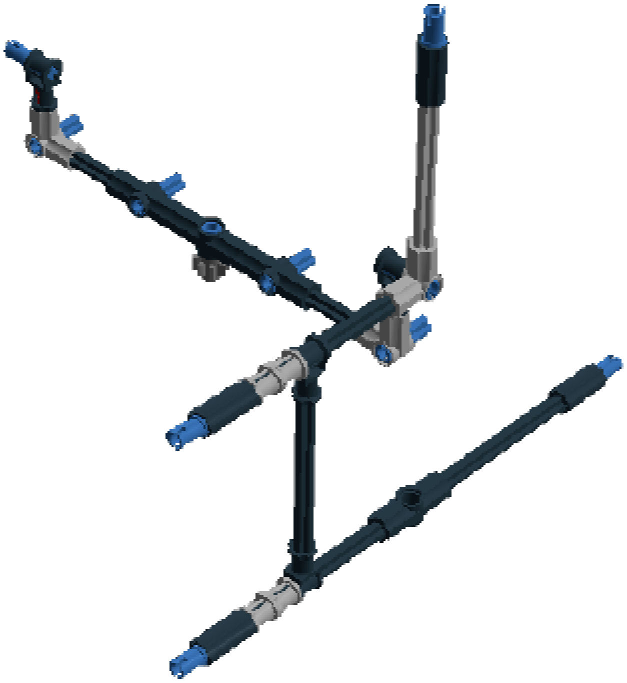

Step 16 of 48

Step 17 of 48

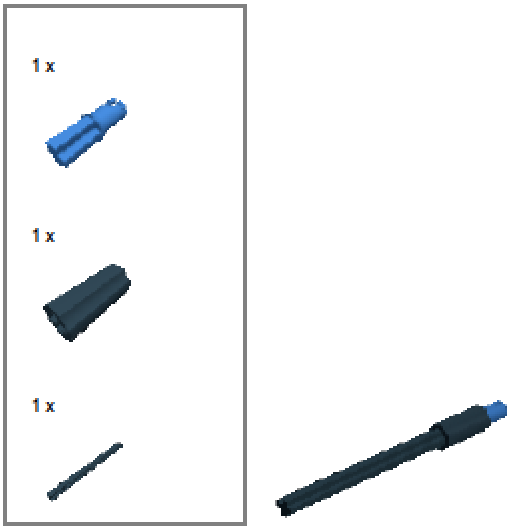

Step 18 of 48

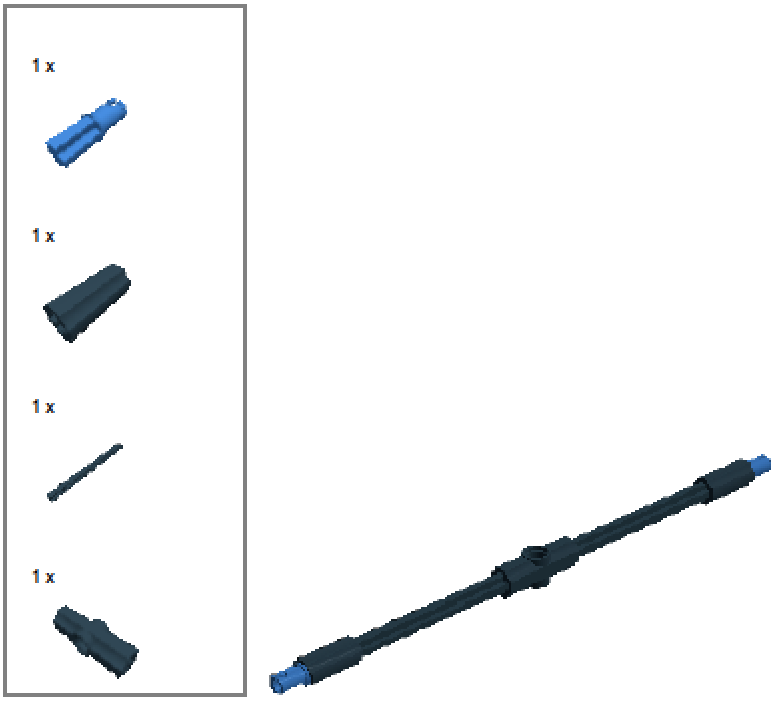

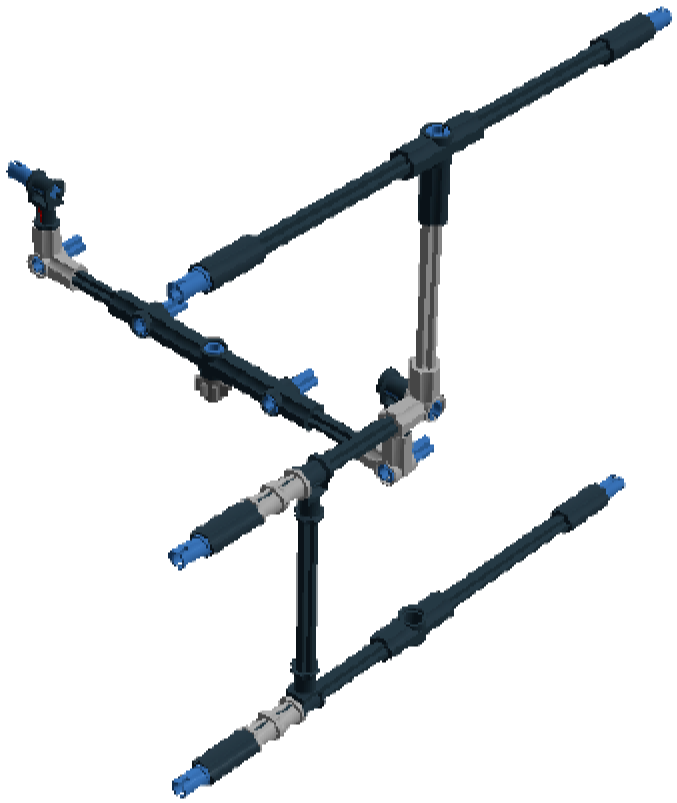

Step 19 of 48

Step 20 of 48

1 x

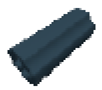

2 x

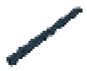

1 x

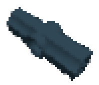

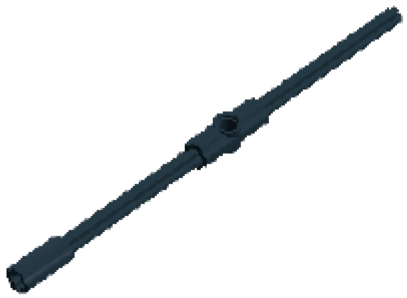

Step 21 of 48

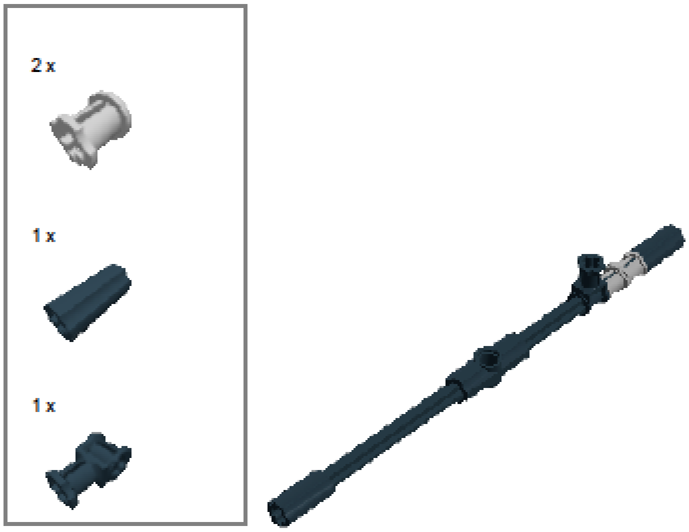

Step 22 of 48

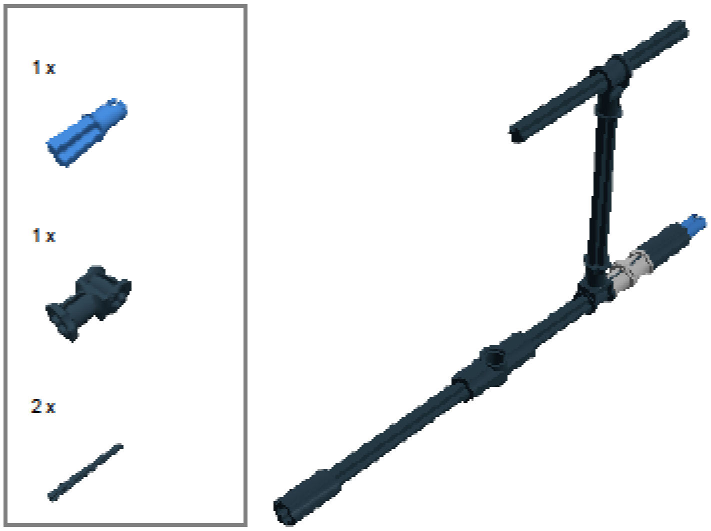

Step 23 of 48

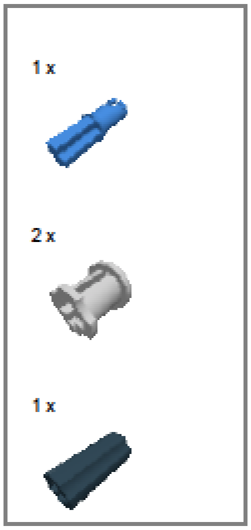

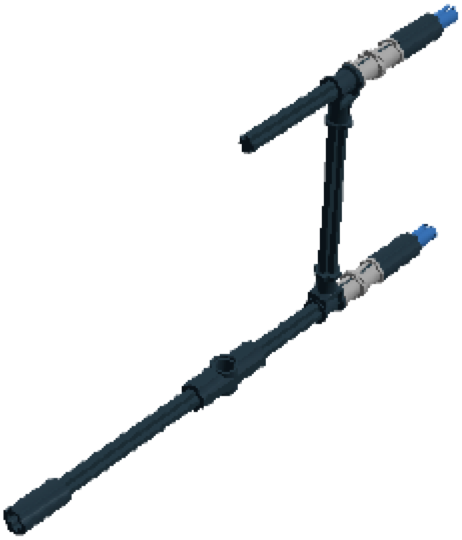

Step 24 of 48

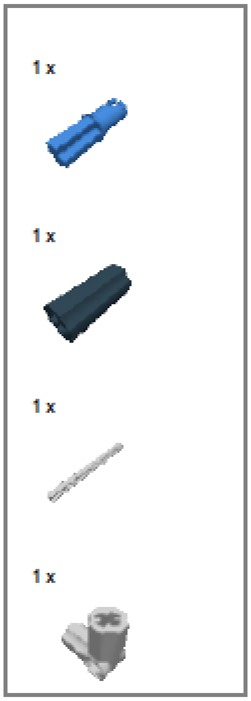

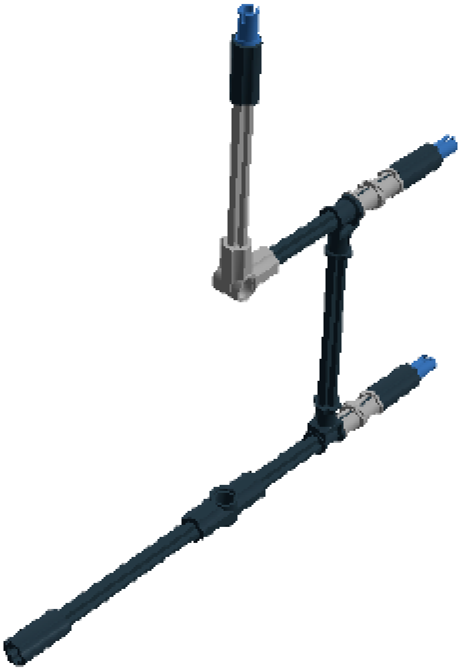

Step 25 of 48

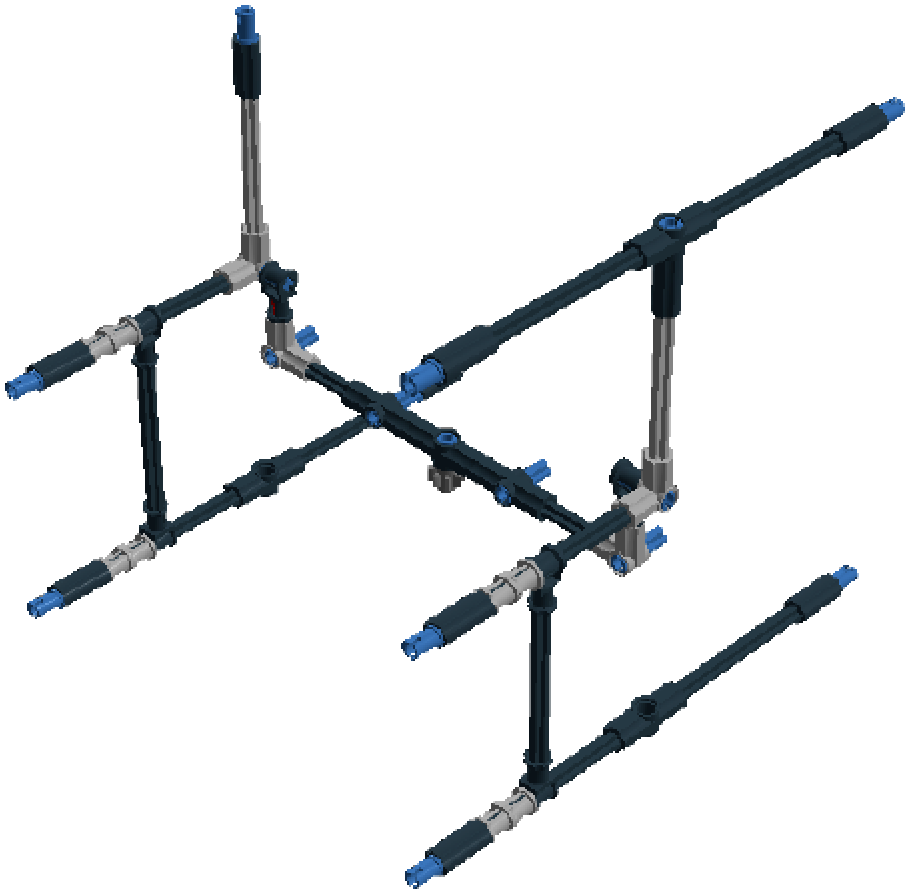

Step 26 of 48

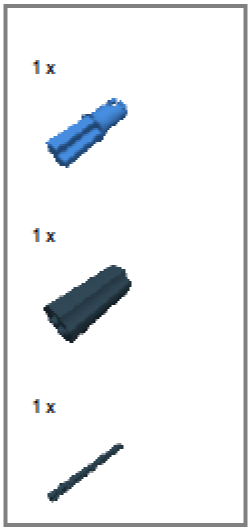

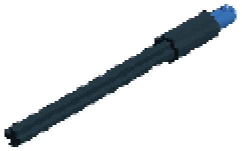

Step 27 of 48

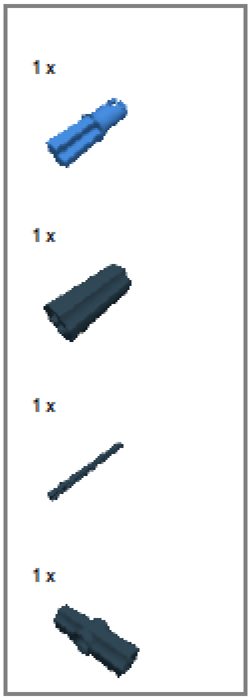

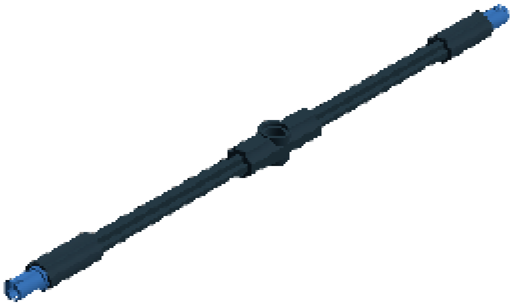

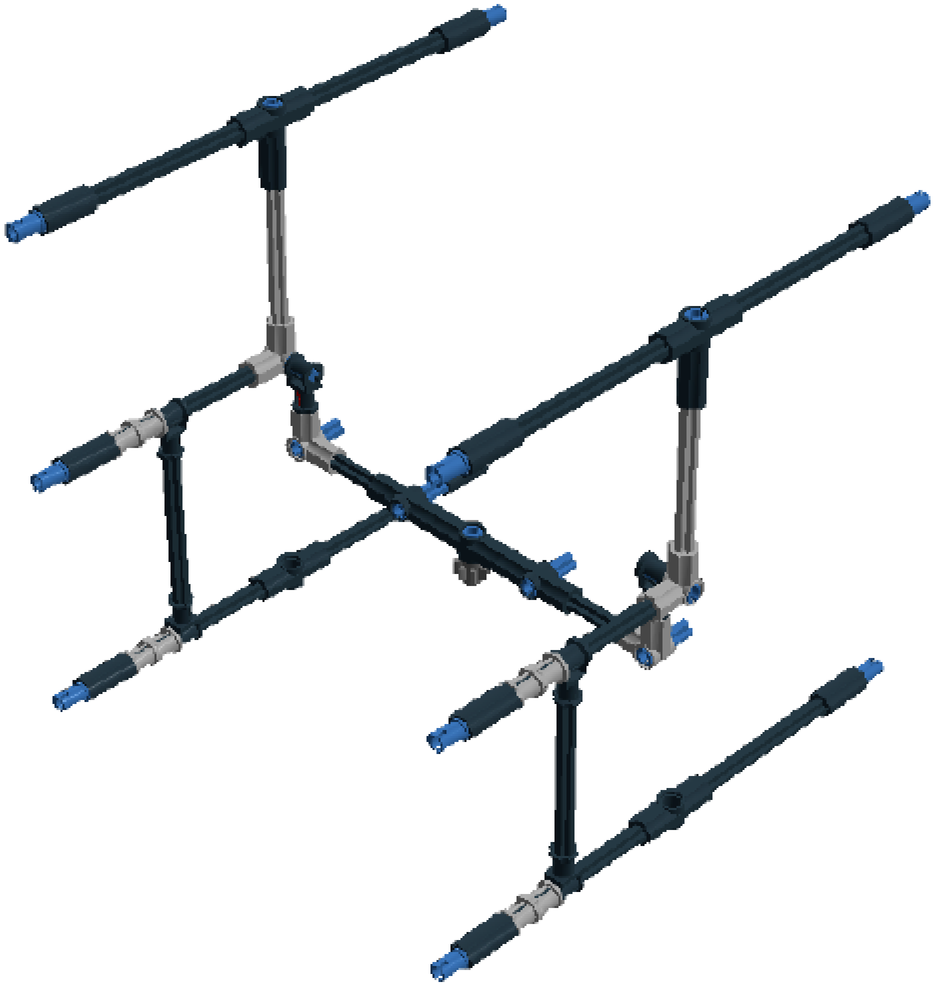

Step 28 of 48

Step 29 of 48

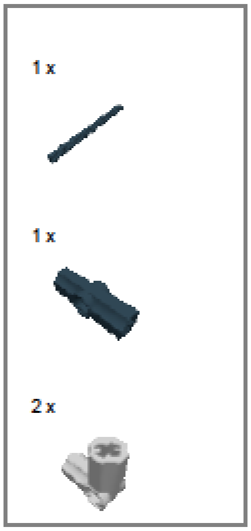

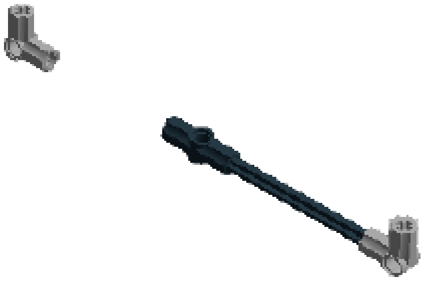

Step 30 of 48

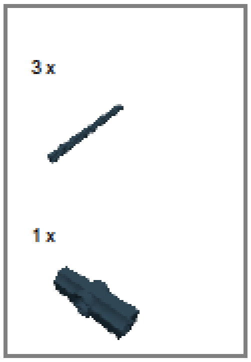

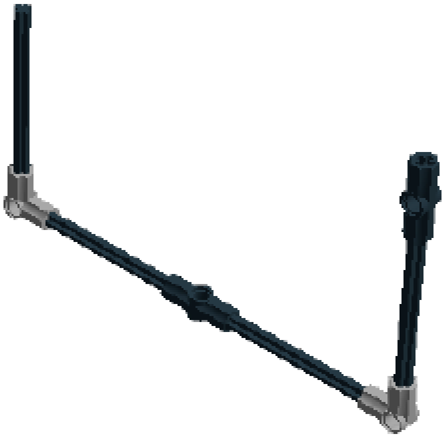

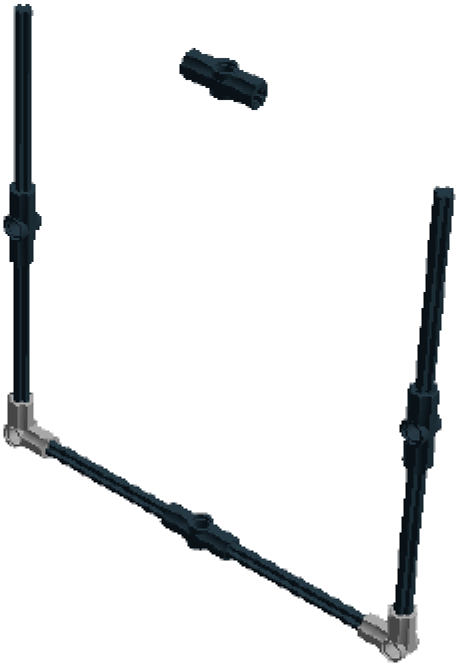

Step 31 of 48

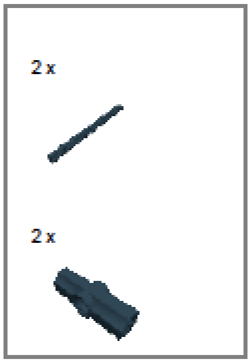

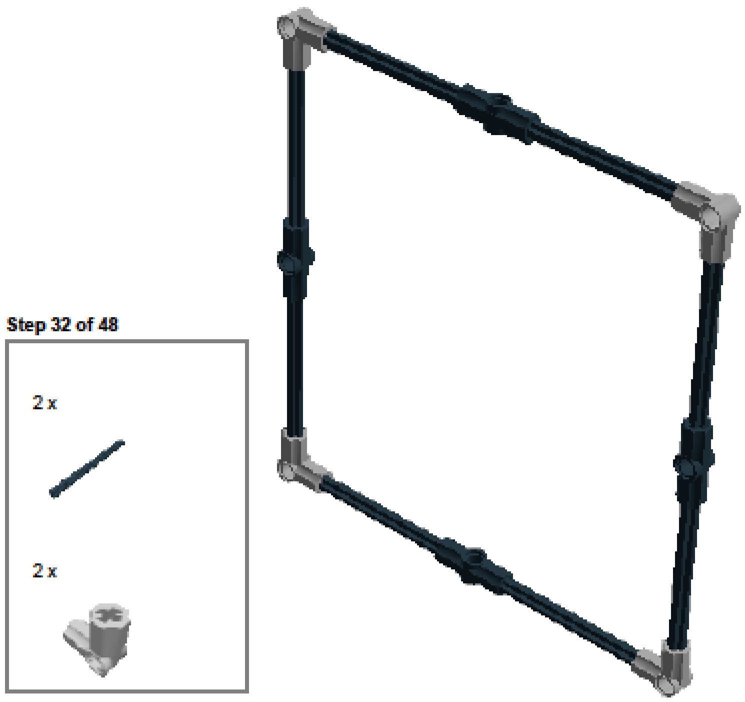
Step 32 of 48
2 x
2 x

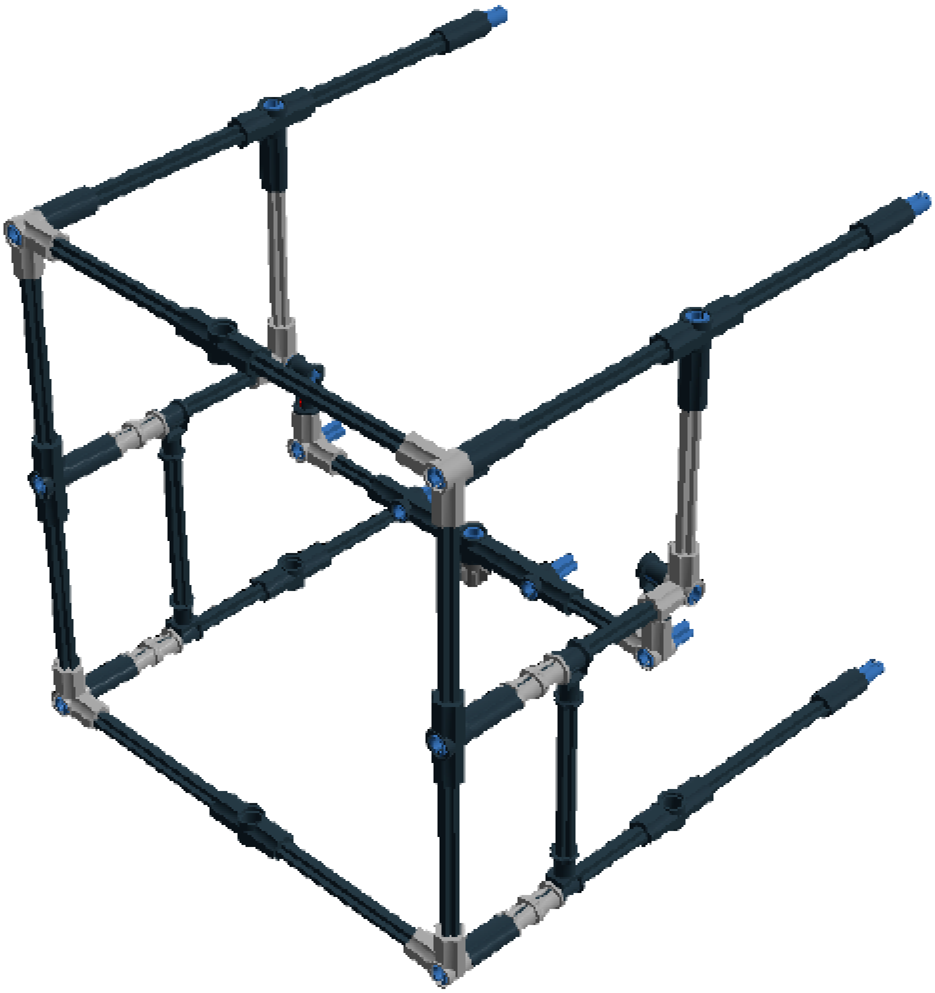

Step 33 of 48

Step 34 of 48

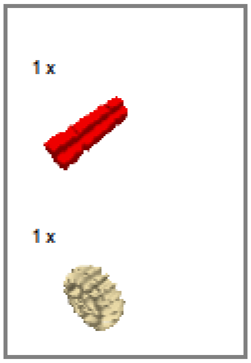

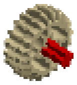

Step 35 of 48

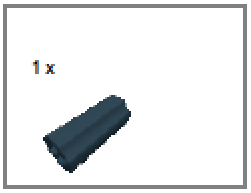

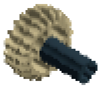

Step 36 of 48

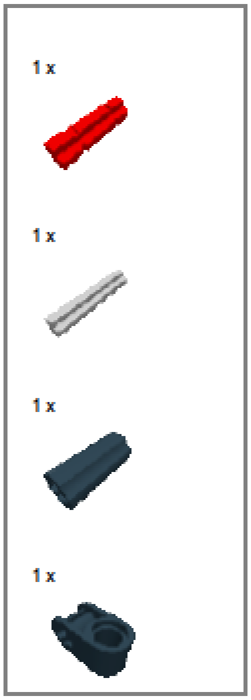

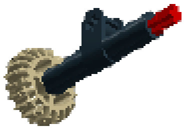

Step 37 of 48

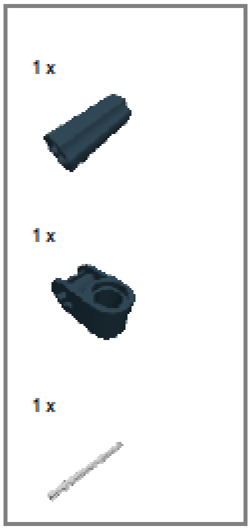

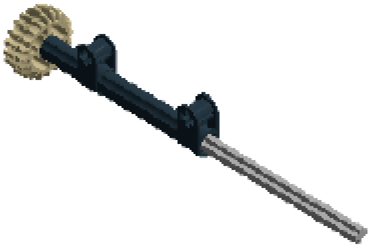

Step 38 of 48

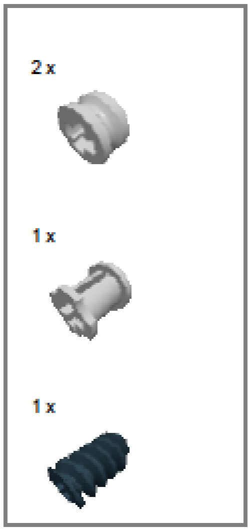

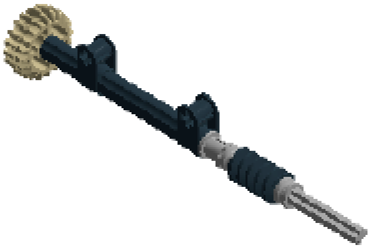

Step 39 of 48

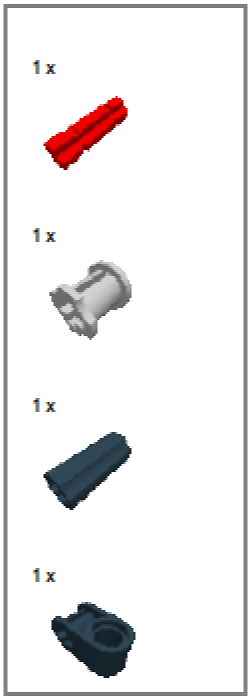

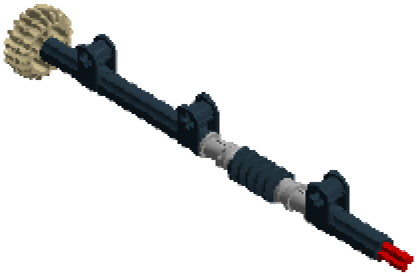

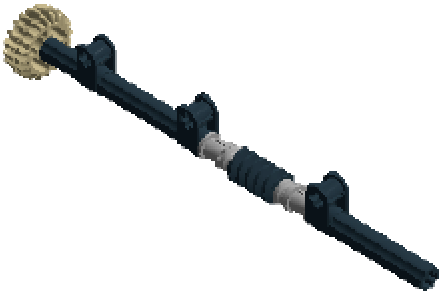

Step 40 of 48

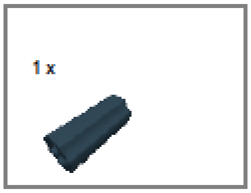

Step 41 of 48

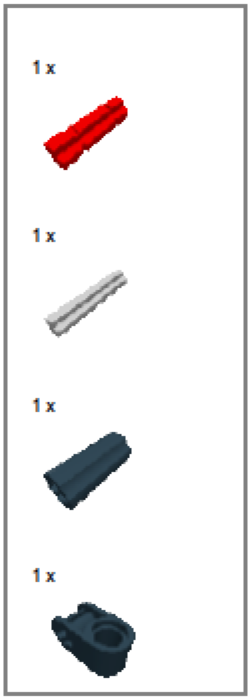

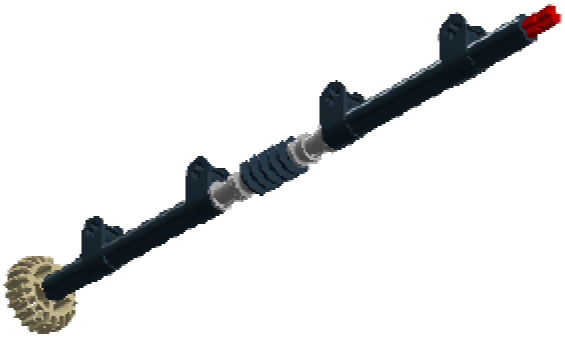

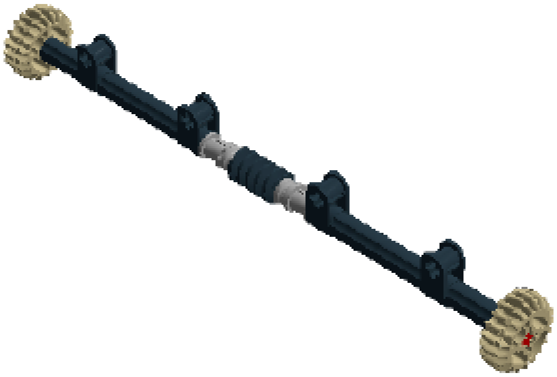

Step 42 of 48

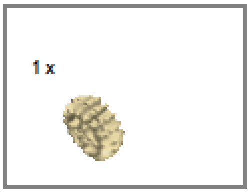

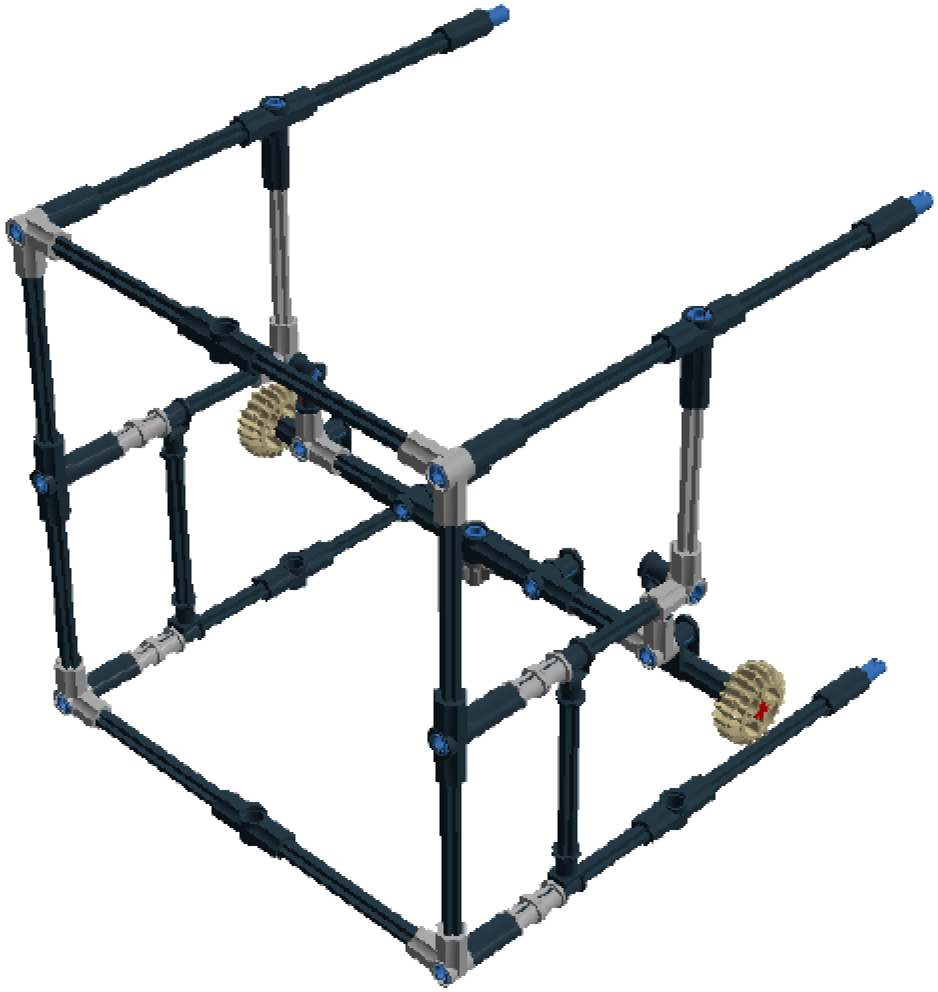

Step 43 of 48

Step 44 of 48

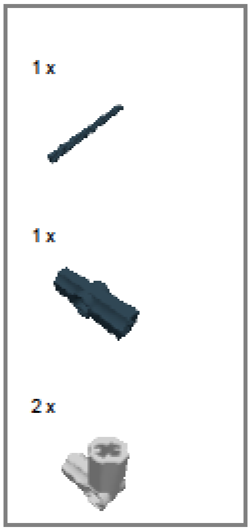

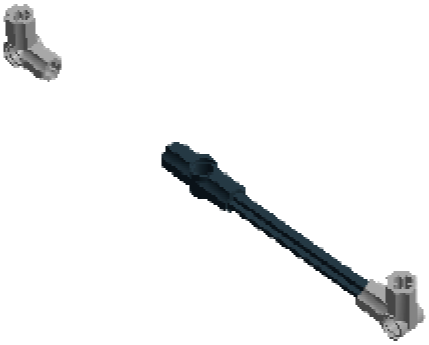

Step 45 of 48

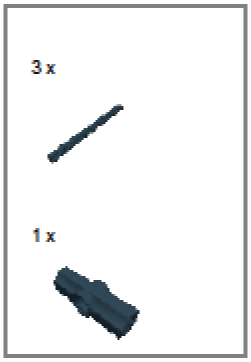

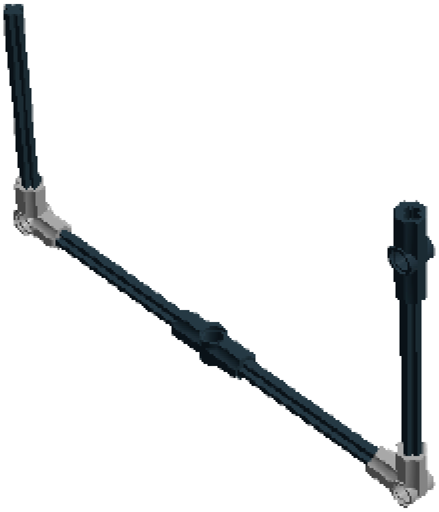

Step 46 of 48

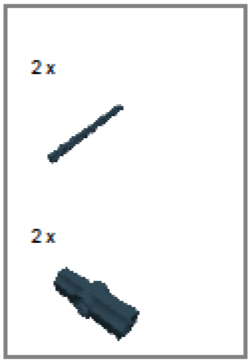

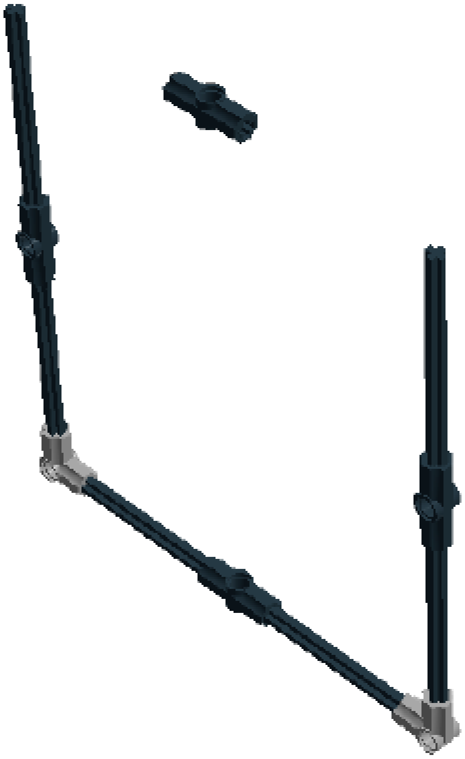

Step 47 of 48

2 x

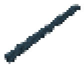

2 x

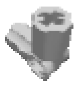

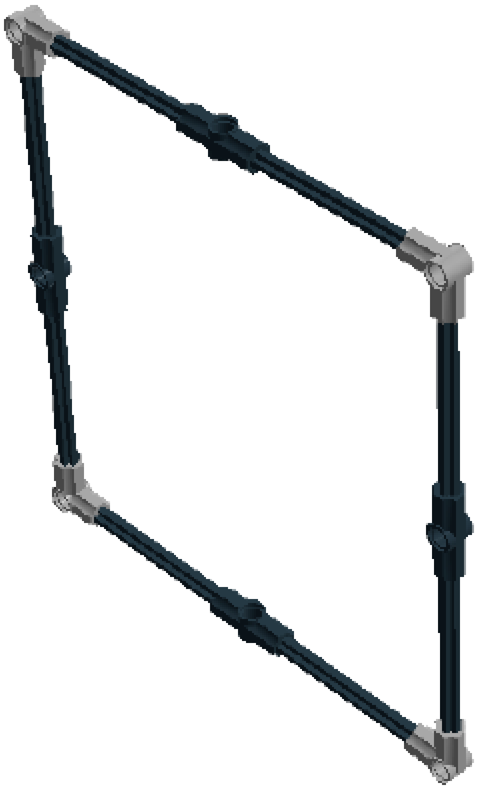

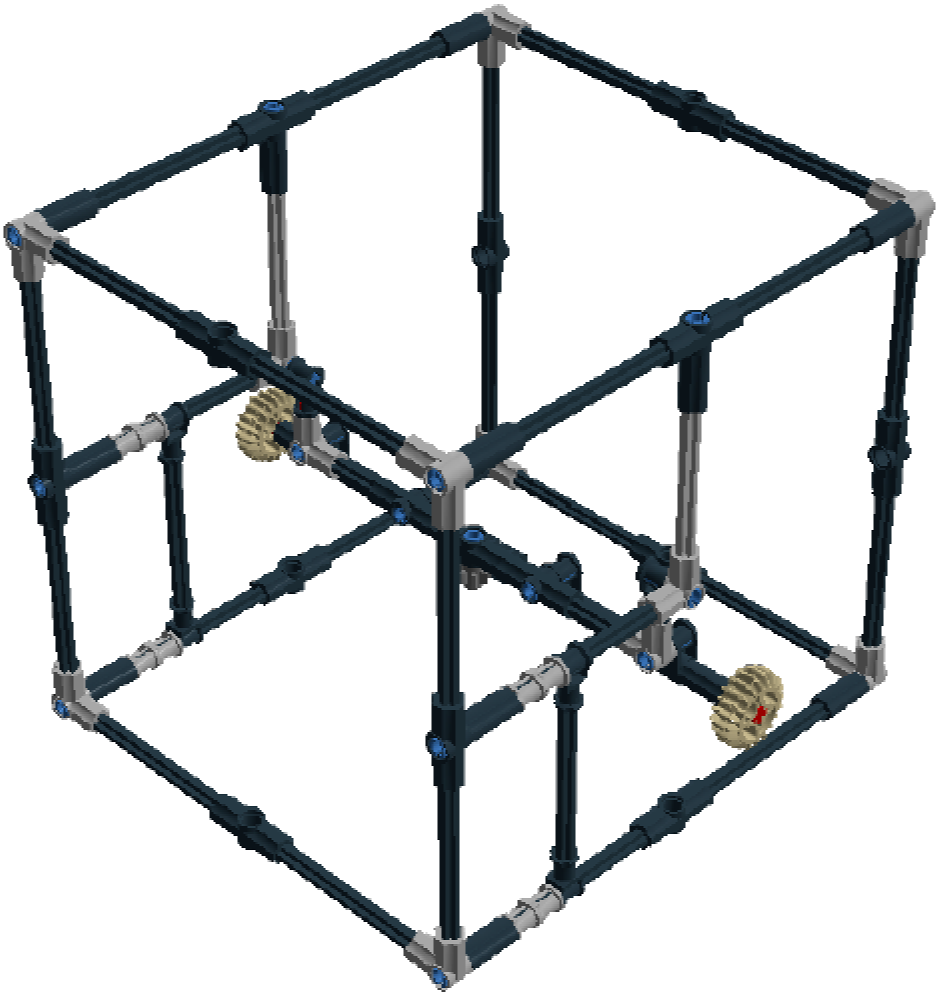

Step 48 of 48

Brick parts with updated design and part IDs

Part IDs represent the same part but in different colours; this can be for aesthetic reasons or in case the part is out of stock on the website.

Image	Name	Design ID	Part IDs	IMp	Micro-	Open-	Giant-	Total
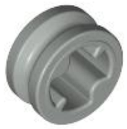	1/2 Bush	32123	4110050<br>4203585<br>4206210<br>4211573<br>4239601<br>4260467<br>4278910	6	2	2	2	12
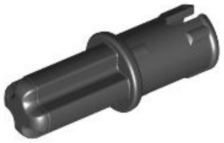	Conn.Bush W.Fric./Cros sale	43093	4184170<br>4184171<br>4206482	14	8	8	18	48
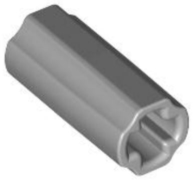	Cross Axle, Extension, 2M	59443	4512360<br>4512363<br>4513174<br>4516546<br>4519010<br>4531751<br>4654508<br>6084612<br>6084613	11	3	6	18	38
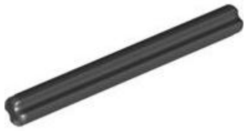	Cross Axle 5M	32073	4114740<br>4141482<br>4166139<br>4211639	1	3	1		5
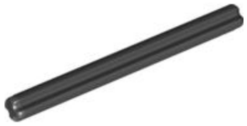	Cross Axle 6M	3706	370626<br>4143263<br>4191526<br>4203604		2	3		5
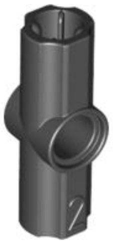	Angle Element, 180 Degrees [2]	32034	4107783<br>4107798<br>4107830<br>4125554<br>4207756<br>4210981<br>4211567<br>4234429<br>6024719	1	3	3	15	22
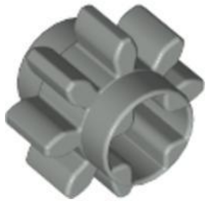	Gear Wheel T=8, M=1	3647	364702<br>4120181<br>4211432<br>4514559	1	1	1	1	4

Image	Name	Design ID	Part IDs	IMp	Micro-	Open-	Giant-	Total
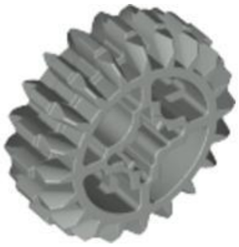	Double Conical Wheel Z20 1M	32269	4141455<br>4177430<br>4205111<br>4211635<br>4514555				2	2
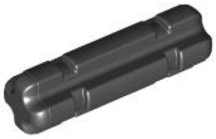	2M Cross Axle W. Groove	32062	4109810<br>4142865<br>4163623	3	7	9	8	27
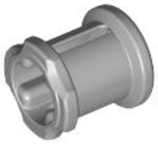	Bush For Cross Axle	6590	4125313<br>4177421<br>4207525<br>4211622<br>4227155<br>4238814	8	0	2	10	20
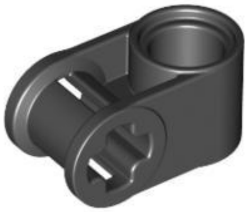	Cross Block 90°	6536	653601<br>653602<br>653626<br>4107078<br>4107766<br>4107799<br>4118881<br>4172101<br>4173600<br>4173601<br>4173658<br>4173659<br>4173661<br>4173663<br>4173665<br>4173666<br>4173667<br>4173668<br>4173669<br>4173670<br>4173987<br>4177420<br>4188298<br>4200936<br>4203896<br>4210851<br>4211775<br>4227273<br>4261453<br>4281196	2	2	2	4	10
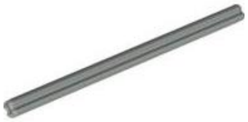	Cross Axle 7M	44294	4183835<br>4211805<br>4267859<br>4268663<br>4631800			1	2	3

Image	Name	Design ID	Part IDs	IMp	Micro-	Open-	Giant-	Total
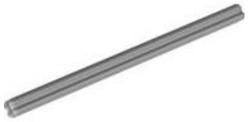	Cross Axle 9M	60485	4535768	4		1	1	6
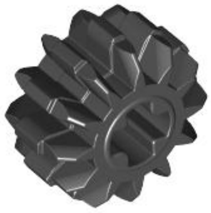	Double Conical Wheel Z12 1M	32270	4141454<br>4177431<br>4211634	1	1	1		3
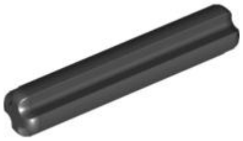	Cross Axle 3M	4519	451926<br>4143467<br>4186007<br>4211815	5	3	1	2	11
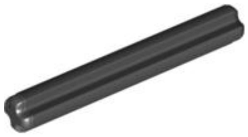	Cross Axle 4M	3705	370526<br>4162390<br>4180062<br>4265797			2	2	4
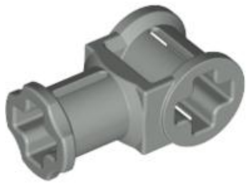	Catch W. Cross Hole	32039	4106469<br>4107081<br>4107800<br>4107801<br>4114233<br>4118897<br>4121616<br>4141419<br>4141420<br>4144128<br>4144294<br>4164128<br>4164357<br>4172048<br>4172110<br>4175548<br>4177427<br>4188140<br>4200926<br>4210669<br>4211553<br>4248903<br>4252466<br>4255607<br>4265704	4	2	2	6	14
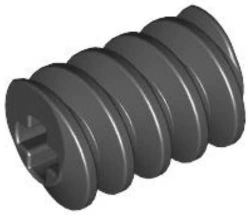	Worm	4716	471602<br>471626<br>4211510<br>6037533	1	1	1	1	4

Image	Name	Design ID	Part IDs	IMp	Micro-	Open-	Giant-	Total
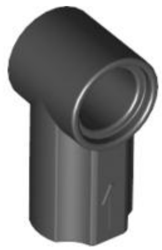	Angle Element, 0 Degrees [1]	32013	4106376<br>4107066<br>4107085<br>4107797<br>4118981<br>4119033<br>4140452<br>4140503<br>4141431<br>4144292<br>4144297<br>4144298<br>4163695<br>4172041<br>4177425<br>4190216<br>4199244<br>4200272<br>4210658<br>4211550<br>4221573<br>4252464<br>4254606<br>4260023<br>4265621<br>4541306	2	2	2		6
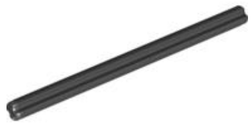	Cross Axle 8M	3707	370726<br>4156302	8			28	36
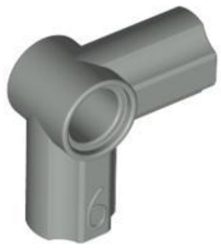	Angle Element, 90 Degrees [6]	32014	4106377<br>4107069<br>4107767<br>4112205<br>4114231<br>4125195<br>4125366<br>4141424<br>4143353<br>4163263<br>4163609<br>4189131<br>4210671<br>4495414<br>4509886<br>4654049<br>6072968	10	8	8	12	38
